# Author Correction: Molecular and phenotypic characteristics of RSV infections in infants during two nirsevimab randomized clinical trials

**DOI:** 10.1038/s41467-024-47421-2

**Published:** 2024-04-08

**Authors:** Bahar Ahani, Kevin M. Tuffy, Anastasia A. Aksyuk, Deidre Wilkins, Michael E. Abram, Ron Dagan, Joseph B. Domachowske, Johnathan D. Guest, Hong Ji, Anna Kushnir, Amanda Leach, Shabir A. Madhi, Vaishali S. Mankad, Eric A. F. Simões, Benjamin Sparklin, Scott D. Speer, Ann Marie Stanley, David E. Tabor, Ulrika Wählby Hamrén, Elizabeth J. Kelly, Tonya Villafana

**Affiliations:** 1 Bioinformatics, Vaccines & Immune Therapies, BioPharmaceuticals R&D, AstraZeneca, Gaithersburg, MD USA; 2 Translational Medicine, Vaccines & Immune Therapies, BioPharmaceuticals R&D, AstraZeneca, Gaithersburg, MD USA; 3 The Shraga Segal Department of Microbiology, Immunology and Genetics, Faculty of Health Sciences of the Ben-Gurion University of the Negev, Beer-Sheva, Israel; 4 State University of New York Upstate Medical University, Syracuse, NY USA; 5 Virology and Vaccine Discovery, Vaccines & Immune Therapies, BioPharmaceuticals R&D, AstraZeneca, Gaithersburg, MD USA; 6 Clinical Development, Vaccines & Immune Therapies, BioPharmaceuticals R&D, AstraZeneca, Gaithersburg, MD USA; 7 South African Medical Research Council Vaccines and Infectious Diseases Analytics Research Unit, Faculty of Health Sciences, University of the Witwatersrand, Johannesburg, South Africa; 8 Clinical Development, Vaccines & Immune Therapies, BioPharmaceuticals R&D, AstraZeneca, Durham, NC USA; 9 University of Colorado School of Medicine and Children’s Hospital Colorado, Aurora, CO USA; 10 Clinical Pharmacology and Quantitative Pharmacology, R&D, AstraZeneca, Gothenburg, Sweden

**Keywords:** Paediatric research, Viral infection, Antibodies, Biological therapy

Correction to: *Nature Communications* 10.1038/s41467-023-40057-8, published online 19 July 2023

The original version of this Article contained an error in Fig. 3b, in which amino acid frequencies at positions 64, 68 and 208 for the ‘Phase 2b’ trial, and at position 204 for the ‘MELODY’ trial were incorrect. The correct version of Fig. 3 is:
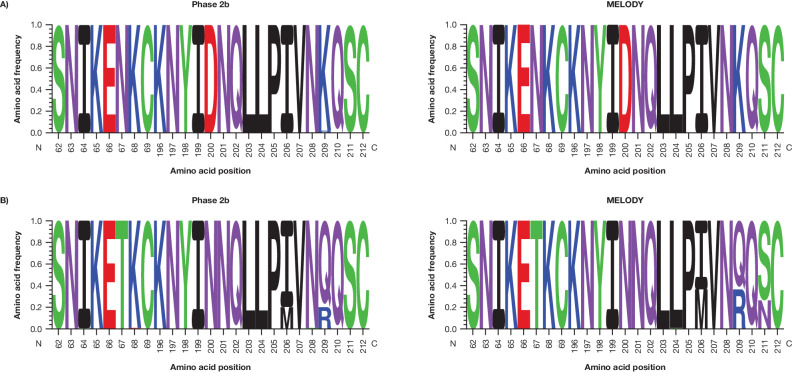


which replaces the previous incorrect version:
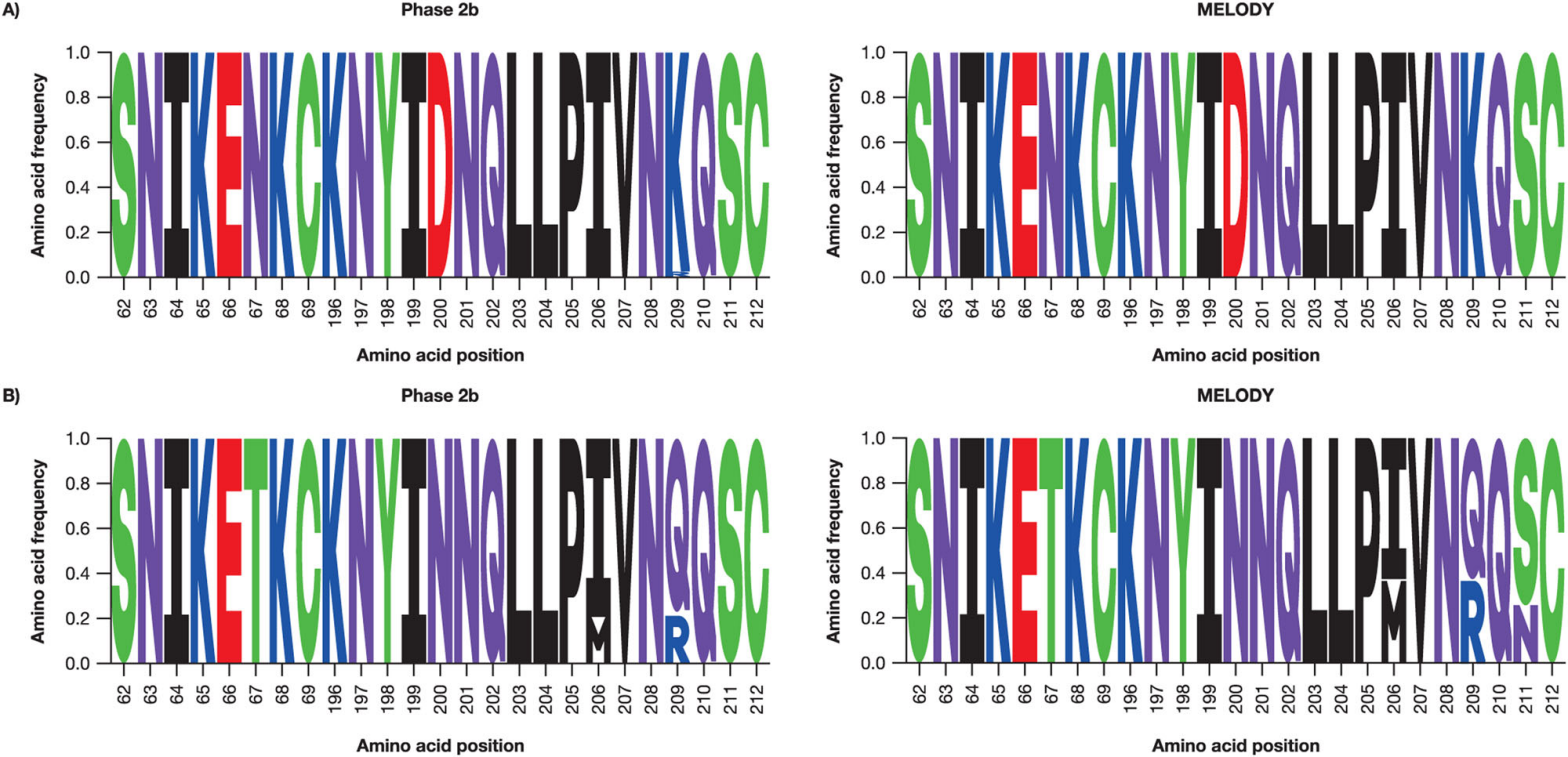


This has been corrected in both the PDF and HTML versions of the Article.

