# Supplementary material for: Molecular and phenotypic characteristics of RSV infections in infants during two nirsevimab randomized clinical trials
Source: Nat Commun. 2023 Jul 19;14:4347. doi: 10.1038/s41467-023-40057-8 (PMC10356750; doi:10.1038/s41467-023-40057-8)
Supplement: Supplementary file 1 — Supplementary Information [file 41467_2023_40057_MOESM1_ESM.pdf]

Supplementary information

Supplementary Note 1. Case narratives of two infants with resistance-associated substitutions

Two infants in the Phase 2b trial experienced a medically attended (MA) respiratory syncytial virus (RSV) lower respiratory tract infection (LRTI), had their infection subtyped as RSV B, and met the primary case definition had three nirsevimab binding substitutions (I64T, N208S, K68E) associated with clinical resistance. Both had a clinical course in line with RSV disease in premature infants in the first year of life.

The first infant (34 weeks gestational age at birth and 5.9 kg at dosing) had an RSV isolate containing the nirsevimab binding substitutions I64T:K68E. No medical history was reported prior to enrollment; medications included vitamin supplementation. The MA RSV LRTI event was reported as a viral pneumonia due to RSV and occurred 118 days post-dose. The infant became symptomatic 2 days after hospital discharge for a preceding human metapneumovirus (hMPV)-associated LRTI (onset 95 days post-dose), and the investigator suspected the RSV LRTI was a nosocomial infection. The infant was re-hospitalized 2 days after symptom onset. A central nasal swab was positive for RSV B and negative for hMPV; local test was positive for RSV. The infant was hospitalized for 12 days and treated with supplemental oxygen in the intensive care unit (ICU) for 9 days, in addition to bronchodilators, systemic steroids, and an antibiotic. The infant recovered and was discharged from the hospital. Over the course of the study, the infant also had six non-RSV MA LRTIs, five of which required hospitalization with one necessitating ICU admission (the above mentioned hMPV-associated LRTI) with continuous positive airway pressure and high flow nasal cannula in conjunction with supplemental oxygen as maximal respiratory support measures. During a hospitalization for an event of viral pneumonia post-dose, the infant underwent diagnostic evaluations for investigation of tracheal aspiration, cystic fibrosis, and immunologic alterations which were ruled out. The infant was diagnosed with bronchomalacia and subsequently maintained on home oxygen after discharge. All LRTI events resolved with the exception of an event of obstructive bronchitis approximately 1 year post-dose; this event was ongoing at the end of the study.

The second infant (34 weeks gestational age at birth and 5.5 kg at dosing) had an RSV isolate containing the nirsevimab binding site substitution N208K. A medical history of RSV-negative bronchitis prior to enrollment was reported; medications included vitamin supplementation. The MA RSV LRTI event was reported as bronchiolitis and occurred 45 days post-dose. A central nasal swab was positive for RSV B and negative for hMPV; local test was positive for RSV. The infant was hospitalized for total of 14 days, comprising ICU admission, mechanical ventilation, and supplemental oxygen. Treatment included bronchodilators, systemic steroids, inhaled epinephrine, and antibiotics. The infant recovered and was discharged. Over the course of the study, the infant had two non-RSV MA LRTIs reported as events of bronchitis, one of which required hospitalization for 6 days, during which the infant was treated with supplemental oxygen, systemic corticosteroids, and bronchodilator. All LRTI events resolved.

Supplementary Table S1 | Summary of case definitions.

Efficacy endpoint	Investigator assesses to have LRTI based on AE coding URTI/LRTI	Clinical sign of LRT involvement[a]	Indicator of disease severity[b]	Meets per protocol definition of MA RSV LRTI	RSV test result[c]	Setting of care
**Primary**						
MA RSV LRTI	Yes	Documented as present	Documented as present	Yes	Positive	Inpatient or outpatient
**Secondary**						
MA RSV LRTI with hospitalization	Yes	Documented as present	Documented as present	Yes	Positive	Inpatient
**Exploratory, RSV respiratory illness**						
RSV unscheduled event	Yes or no	May or not be present	May or not be present	No	Positive	Inpatient or Outpatient
Non-protocol defined LRTI outpatient event	Yes	May or not be present[d]	May or not be present[d]	No	Positive	Outpatient
Hospitalization due to RSV Non-LRTI	Yes or no	May or not be present[d]	May or not be present[d]	No	Positive	Inpatient

[a]Includes at least one of the following: Rhonchi, rales, crackles, wheeze.

[b]Includes at least one of the following: Hypoxemia, acute hypoxic or ventilatory failure; new onset apnea, nasal flaring, retractions, grunting, dehydration due to respiratory distress.

[c]Confirmed from central test by RT-PCR.

[d]Includes cases where insufficient documentation of the event was available to the investigator.

AE, adverse event; LRT, lower respiratory tract; LRTI, lower respiratory tract infection; MA, medically attended; RSV, respiratory syncytial virus; RT-PCR, reverse transcriptase polymerase chain reaction; URTI, upper respiratory tract infection.

Supplementary Table S2 | Prevalence and frequency of all major variant substitutions observed for cases that met the primary case definition in the Phase 2b and MELODY trials.

Sampling period	Amino acid substitutions in RSV F	Prevalence in surveillance studies (%)	Frequency		
			**Placebo n (%)**	**Nirsevimab (n (%)**	**Total n (%)**
		**Phase 2b – RSV A**			
**Through 150 days post-dose**			**n = 22**	**n = 11**	**n = 33**
	T13A	4.66	0 (0)	1 (9.1)	1 (3)
	T13I	0.07	1 (4.5)	0 (0)	1 (3)
	I14V	0.07	1 (4.5)	0 (0)	1 (3)
	A17V	0.21	1 (4.5)	1 (9.1)	2 (6.1)
	C21S	NA	1 (4.5)	0 (0)	1 (3)
	F22I	0.07	1 (4.5)	0 (0)	1 (3)
	A23T	5.32	2 (9.1)	1 (9.1)	3 (9.1)
	S25N	0.42	0 (0)	1 (9.1)	1 (3)
	G71S	NA	1 (4.5)	0 (0)	1 (3)
	V76I	NA	0 (0)	1 (9.1)	1 (3)
	S99N	0.1	1 (4.5)	0 (0)	1 (3)
	A102S	0.03	1 (4.5)	1 (9.1)	2 (6.1)
	N116Y	NA	1 (4.5)	0 (0)	1 (3)
	N120S	0.35	1 (4.5)	0 (0)	1 (3)
	K123E	0.03	0 (0)	1 (9.1)	1 (3)
	V127A	1.15	0 (0)	1 (9.1)	1 (3)
	S213R	0.1	0 (0)	1 (9.1)	1 (3)
	K419E	0.1	1 (4.5)	1 (9.1)	2 (6.1)
	**Nirsevimab binding site**		None		
	**Palivizumab binding site**		None		
**151–360 days post-dose**			**n = 2**	**n = 3**	**n = 5**
	I14V	0.07	0 (0)	1 (33.3)	1 (20)
	A23T	5.32	0 (0)	2 (66.7)	2 (40)

	R113K	0.1	1 (50)	0 (0)	1 (20)
	Y117H	1.39	1 (50)	0 (0)	1 (20)
	S190N	0.07	0 (0)	1 (33.3)	1 (20)
	**Nirsevimab binding site**		None		
	**Palivizumab binding site**		None		
**Phase 2b – RSV B**					
**Through 150 days post-dose**			**n = 22**	**n = 14**	**n = 36**
	L4P	2.25	1 (4.5)	0 (0)	1 (2.8)
	F12L	7.21	1 (4.5)	2 (14.3)	3 (8.3)
	F15I	NA	1 (4.5)	0 (0)	1 (2.8)
	F15L	99.57	21 (95.5)	14 (100)	35 (97.2)
	A16T	1.21	1 (4.5)	0 (0)	1 (2.8)
	A16V	1.57	1 (4.5)	0 (0)	1 (2.8)
	I64T	NA	0 (0)	1 (7.1)	1 (2.8)
	K68E	NA	0 (0)	1 (7.1)	1 (2.8)
	A103V	98.5	22 (100)	14 (100)	36 (100)
	T118I	0.07	1 (4.5)	0 (0)	1 (2.8)
	V127I	0.07	0 (0)	1 (7.1)	1 (2.8)
	L172Q	98.61	22 (100)	14 (100)	36 (100)
	S173L	98.36	22 (100)	14 (100)	36 (100)
	K191R	68.61	8 (36.4)	4 (28.6)	12 (33.3)
	I206M	68.89	8 (36.4)	4 (28.6)	12 (33.3)
	N208S	NA	0 (0)	1 (7.1)	1 (2.8)
	Q209R	68.18	8 (36.4)	4 (28.6)	12 (33.3)
	S276N	7.14	2 (9.1)	2 (14.3)	4 (11.1)
	K327R	0.11	0 (0)	1 (7.1)	1 (2.8)
	E463D	7	1 (4.5)	2 (14.3)	3 (8.3)

	I527M	0.04	1 (4.5)	0 (0)	1 (2.8)
	A543T	0.07	1 (4.5)	0 (0)	1 (2.8)
	K574N	0.07	0 (0)	1 (7.1)	1 (2.8)
	**Nirsevimab binding site**				
	I64T	NA	0 (0)	1 (7.1)	1 (2.8)
	K68E	NA	0 (0)	1 (7.1)	1 (2.8)
	I206M	68.89	8 (36.4)	4 (28.6)	12 (33.3)
	N208S	NA	0 (0)	1 (7.1)	1 (2.8)
	Q209R	68.18	8 (36.4)	4 (28.6)	12 (33.3)
	**Palivizumab binding site**		None		
**151–360 days post-dose**			**n = 2**	**n = 3**	**n = 5**
	F15L	99.57	2 (100)	3 (100)	5 (100)
	A16V	1.57	0 (0)	1 (33.3)	1 (20)
	Y33F	0.07	0 (0)	1 (33.3)	1 (20)
	A103V	98.5	2 (100)	3 (100)	5 (100)
	V127I	0.07	1 (50)	0 (0)	1 (20)
	L172Q	98.61	2 (100)	3 (100)	5 (100)
	S173L	98.36	2 (100)	3 (100)	5 (100)
	K191R	68.61	1 (50)	0 (0)	1 (20)
	I206M	68.89	1 (50)	0 (0)	1 (20)
	Q209R	68.18	1 (50)	0 (0)	1 (20)
	**Nirsevimab binding site**				
	I206M	68.89	1 (50)	0 (0)	1 (20)
	Q209R	68.18	1 (50)	0 (0)	1 (20)
	**Palivizumab binding site**		None		

MELODY full cohort – RSV A				
**Through 150 days post-dose**		**n = 23**	**n = 13**	**n = 36**
L6H	0.14	1 (4.3)	0 (0)	1 (2.8)
T8I	0.45	0 (0)	1 (7.7)	1 (2.8)
T12I	6.75	6 (26.1)	2 (15.4)	8 (22.2)
T13A	4.66	2 (8.7)	1 (7.7)	3 (8.3)
L15F	2.09	0 (0)	2 (15.4)	2 (5.6)
L20F	0.77	1 (4.3)	0 (0)	1 (2.8)
A23T	5.32	1 (4.3)	0 (0)	1 (2.8)
A103T	1.91	1 (4.3)	1 (7.7)	2 (5.6)
A107T	1.04	0 (0)	2 (15.4)	2 (5.6)
F114S	0.94	2 (8.7)	0 (0)	2 (5.6)
M115T	0.87	1 (4.3)	0 (0)	1 (2.8)
L119F	0.07	0 (0)	1 (7.7)	1 (2.8)
T122A	11.58	3 (13)	5 (38.5)	8 (22.2)
K123Q	3.62	0 (0)	1 (7.7)	1 (2.8)
V127A	1.15	1 (4.3)	0 (0)	1 (2.8)
V127I	0.21	1 (4.3)	0 (0)	1 (2.8)
V144I	0.1	1 (4.3)	0 (0)	1 (2.8)
T245N	NA	1 (4.3)	0 (0)	1 (2.8)
S255N	0.03	0 (0)	1 (7.7)	1 (2.8)
S276N	2.05	2 (8.7)	0 (0)	2 (5.6)
Q354R	NA	0 (0)	1 (7.7)	1 (2.8)
I384T	3.76	0 (0)	1 (7.7)	1 (2.8)
V406I	0.66	1 (4.3)	0 (0)	1 (2.8)
K419E	0.1	0 (0)	1 (7.7)	1 (2.8)

	D479N	0.1	0 (0)	1 (7.7)	1 (2.8)
	D486N	NA	1 (4.3)	0 (0)	1 (2.8)
	**Nirsevimab binding site**		None		
	**Palivizumab binding site**		None		
**151–360 days post-dose**			**n = 6**	**n = 7**	**n = 13**
	A23S	0.1	0 (0)	1 (14.3)	1 (7.7)
	F114S	0.94	0 (0)	1 (14.3)	1 (7.7)
	T122A	11.58	6 (100)	6 (85.7)	12 (92.3)
	K123Q	3.62	6 (100)	5 (71.4)	11 (84.6)
	I384T	3.76	6 (100)	5 (71.4)	11 (84.6)
	D562N	0.1	1 (16.7)	0 (0)	1 (7.7)
	**Nirsevimab binding site**		None		
	**Palivizumab binding site**		None		
**361–511 days post-dose**			**n = 1**	**n = 4**	**n = 5**
	T12I	6.75	0 (0)	1 (25)	1 (20)
	A23S	0.1	1 (100)	2 (50)	3 (60)
	T122A	11.58	1 (100)	3 (75)	4 (80)
	K123Q	3.62	0 (0)	1 (25)	1 (20)
	E378D	0.03	1 (100)	0 (0)	1 (20)
	I384T	3.76	0 (0)	1 (25)	1 (20)
	S443T	0.07	0 (0)	1 (25)	1 (20)
	K551R	0.03	0 (0)	1 (25)	1 (20)
	**Nirsevimab binding site**		None		
	**Palivizumab binding site**		None		

MELODY full cohort – RSV B					
**Through 150 days post-dose**			**n = 29**	**n = 10**	**n = 39**
	I11L	NA	1 (3.4)	0 (0)	1 (2.6)
	F12I	0.07	7 (24.1)	5 (50)	12 (30.8)
	F15L	99.57	26 (89.7)	10 (100)	36 (92.3)
	A16V	1.57	1 (3.4)	0 (0)	1 (2.6)
	N18S	0.21	1 (3.4)	0 (0)	1 (2.6)
	L22P	1.68	1 (3.4)	0 (0)	1 (2.6)
	T91N	NA	1 (3.4)	0 (0)	1 (2.6)
	A103V	98.5	29 (100)	10 (100)	39 (100)
	A111V	0.07	1 (3.4)	0 (0)	1 (2.6)
	N116S	0.21	1 (3.4)	0 (0)	1 (2.6)
	L172Q	98.61	29 (100)	10 (100)	39 (100)
	S173L	98.36	28 (96.6)	8 (80)	36 (92.3)
	S190N	2.79	22 (75.9)	9 (90)	31 (79.5)
	K191R	68.61	28 (96.6)	10 (100)	38 (97.4)
	L204S	NA	0 (0)	1 (10)	1 (2.6)
	I206M	68.89	28 (96.6)	10 (100)	38 (97.4)
	Q209R	68.18	28 (96.6)	10 (100)	38 (97.4)
	S211N	1.14	22 (75.9)	9 (90)	31 (79.5)
	V239I	0.14	1 (3.4)	0 (0)	1 (2.6)
	K272R	0.04	0 (0)	1 (10)	1 (2.6)
	K327E	0.04	1 (3.4)	0 (0)	1 (2.6)
	V365I	NA	1 (3.4)	0 (0)	1 (2.6)
	P376S	NA	1 (3.4)	0 (0)	1 (2.6)
	S389P	1.07	22 (75.9)	9 (90)	31 (79.5)

	S436P	NA	1 (3.4)	0 (0)	1 (2.6)
	T522A	0.07	1 (3.4)	0 (0)	1 (2.6)
	A529V	0.93	1 (3.4)	0 (0)	1 (2.6)
	**Nirsevimab binding site**				
	L204S	NA	0 (0)	1 (10)	1 (2.6)
	I206M	68.89	28 (96.6)	10 (100)	38 (97.4)
	Q209R	68.18	28 (96.6)	10 (100)	38 (97.4)
	S211N	1.14	22 (75.9)	9 (90)	31 (79.5)
	**Palivizumab binding site**				
	K272R	0.04	0 (0)	1 (10)	1 (2.6)
**151–360 days post-dose**			**n = 0**	**n = 6**	**n = 6**
	F15L	99.57	0 (0)	6 (100)	6 (100)
	A103V	98.5	0 (0)	6 (100)	6 (100)
	L172Q	98.61	0 (0)	4 (66.7)	4 (66.7)
	S173L	98.36	0 (0)	4 (66.7)	4 (66.7)
	S190N	2.79	0 (0)	1 (16.7)	1 (16.7)
	K191R	68.61	0 (0)	4 (66.7)	4 (66.7)
	I206M	68.89	0 (0)	4 (66.7)	4 (66.7)
	Q209R	68.18	0 (0)	4 (66.7)	4 (66.7)
	S211N	1.14	0 (0)	1 (16.7)	1 (16.7)
	S330T	NA	0 (0)	1 (16.7)	1 (16.7)
	S389P	1.07	0 (0)	2 (33.3)	2 (33.3)
	**Nirsevimab binding site**				
	I206M	68.89	0 (0)	4 (66.7)	4 (66.7)
	Q209R	68.18	0 (0)	4 (66.7)	4 (66.7)
	S211N	1.14	0 (0)	1 (16.7)	1 (16.7)

	**Palivizumab binding site**		None		
**361–511 days post-dose**			**n = 1**	**n = 3**	**n = 4**
	F15L	99.57	1 (100)	3 (100)	4 (100)
	A103V	98.5	1 (100)	3 (100)	4 (100)
	N120S	0.11	0 (0)	1 (33.3)	1 (25)
	L172Q	98.61	1 (100)	3 (100)	4 (100)
	S173L	98.36	1 (100)	3 (100)	4 (100)
	K191R	68.61	1 (100)	3 (100)	4 (100)
	I206M	68.89	1 (100)	3 (100)	4 (100)
	Q209R	68.18	1 (100)	3 (100)	4 (100)
	S330T	NA	1 (100)	0 (0)	1 (25)
	**Nirsevimab binding site**				
	I206M	68.89	1 (100)	3 (100)	4 (100)
	Q209R	68.18	1 (100)	3 (100)	4 (100)
	**Palivizumab binding site**		None		

Major variants had ≥25% MAF in surveillance studies. Site Ø=AAs 62–96 and 195–227; Site II=AAs 254–277; non-EC regions: signal peptide=AAs 1–23; p27=AAs 110–136; transmembrane and intracellular domains=AAs 525–574; nirsevimab binding site=AAs 62–69 and 196–212; palivizumab binding site=AAs 262–275.

AA, amino acid; EC, extracellular; MAF, molecular allele frequency; NA, not applicable (i.e. not observed); RSV, respiratory syncytial virus.

Supplementary Table S3 | Prevalence and frequency of all major variant substitutions observed for cases that met the secondary case definition in the Phase 2b and MELODY trials.

Sampling period	Amino acid substitutions in RSV F	Prevalence in surveillance studies (%)	Frequency		
			Placebo n (%)	Nirsevimab n (%)	Total n (%)
		**Phase 2b – RSV A**			
**Through 150 days post-dose**			**n = 12**	**n = 5**	**n = 17**
	T13A	4.66	0 (0)	1 (20)	1 (5.9)
	A17V	0.21	1 (8.3)	0 (0)	1 (5.9)
	F22I	0.07	1 (8.3)	0 (0)	1 (5.9)
	A23T	5.32	1 (8.3)	1 (20)	2 (11.8)
	A102S	0.03	1 (8.3)	1 (20)	2 (11.8)
	K123E	0.03	0 (0)	1 (20)	1 (5.9)
	S213R	0.1	0 (0)	1 (20)	1 (5.9)
	K419E	0.1	1 (8.3)	1 (20)	2 (11.8)
	**Nirsevimab binding site**		None		
	**Palivizumab binding site**		None		
**151–360 days post-dose**	R113K	0.1	1 (100)	0 (0)	1 (100)
	Y117H	1.39	1 (100)	0 (0)	1 (100)
	**Nirsevimab binding site**		None		
	**Palivizumab binding site**		None		
		**Phase 2b – RSV B**			
**Through 150 days post-dose**			**n = 8**	**n = 3**	**n = 11**
	F12L	7.21	0 (0)	1 (33.3)	1 (9.1)
	F15I	NA	1 (12.5)	0 (0)	1 (9.1)
	F15L	99.57	7 (87.5)	3 (100)	10 (90.9)

	A16T	1.21	1 (12.5)	0 (0)	1 (9.1)
	A16V	1.57	1 (12.5)	0 (0)	1 (9.1)
	I64T	NA	0 (0)	1 (33.3)	1 (9.1)
	K68E	NA	0 (0)	1 (33.3)	1 (9.1)
	A103V	98.5	8 (100)	3 (100)	11 (100)
	L172Q	98.61	8 (100)	3 (100)	11 (100)
	S173L	98.36	8 (100)	3 (100)	11 (100)
	K191R	68.61	1 (12.5)	1 (33.3)	2 (18.2)
	I206M	68.89	1 (12.5)	1 (33.3)	2 (18.2)
	N208S	NA	0 (0)	1 (33.3)	1 (9.1)
	Q209R	68.18	1 (12.5)	1 (33.3)	2 (18.2)
	S276N	7.14	1 (12.5)	1 (33.3)	2 (18.2)
	K327R	0.11	0 (0)	1 (33.3)	1 (9.1)
	E463D	7	0 (0)	1 (33.3)	1 (9.1)
	K574N	0.07	0 (0)	1 (33.3)	1 (9.1)
	**Nirsevimab binding site**				
	I64T	NA	0 (0)	1 (33.3)	1 (9.1)
	K68E	NA	0 (0)	1 (33.3)	1 (9.1)
	I206M	68.89	1 (12.5)	1 (33.3)	2 (18.2)
	N208S	NA	0 (0)	1 (33.3)	1 (9.1)
	Q209R	68.18	1 (12.5)	1 (33.3)	2 (18.2)
	**Palivizumab binding site**		None		
**151–360 days post-dose**			**n = 1**	**n = 1**	**n = 2**
	F15L	99.57	1 (100)	1 (100)	2 (100)
	A103V	98.5	1 (100)	1 (100)	2 (100)
	V127I	0.07	1 (100)	0 (0)	1 (50)

	L172Q	98.61	1 (100)	1 (100)	2 (100)
	S173L	98.36	1 (100)	1 (100)	2 (100)
	K191R	68.61	1 (100)	0 (0)	1 (50)
	I206M	68.89	1 (100)	0 (0)	1 (50)
	Q209R	68.18	1 (100)	0 (0)	1 (50)
	**Nirsevimab binding site**				
	I206M	68.89	1 (100)	0 (0)	1 (50)
	Q209R	68.18	1 (100)	0 (0)	1 (50)
	**Palivizumab binding site**		None		
**MELODY full cohort – RSV A**					
**Through 150 days post-dose**			**n = 8**	**n = 7**	**n = 15**
	T8I	0.45	0 (0)	1 (14.3)	1 (6.7)
	T12I	6.75	2 (25)	1 (14.3)	3 (20)
	T13A	4.66	1 (12.5)	1 (14.3)	2 (13.3)
	L15F	2.09	0 (0)	1 (14.3)	1 (6.7)
	A103T	1.91	1 (12.5)	0 (0)	1 (6.7)
	A107T	1.04	0 (0)	2 (28.6)	2 (13.3)
	F114S	0.94	1 (12.5)	0 (0)	1 (6.7)
	T122A	11.58	1 (12.5)	2 (28.6)	3 (20)
	V127A	1.15	1 (12.5)	0 (0)	1 (6.7)
	S276N	2.05	1 (12.5)	0 (0)	1 (6.7)
	K419E	0.1	0 (0)	1 (14.3)	1 (6.7)
	D486N	NA	1 (12.5)	0 (0)	1 (6.7)
	**Nirsevimab binding site**		None		
	**Palivizumab binding site**		None		

**151–360 days post-dose**			**n = 2**	**n = 1**	**n = 3**
	T122A	11.58	2 (100)	1 (100)	3 (100)
	K123Q	3.62	2 (100)	1 (100)	3 (100)
	I384T	3.76	2 (100)	1 (100)	3 (100)
	**Nirsevimab binding site**		None		
	**Palivizumab binding site**		None		
**361–511 days post-dose**	**Nirsevimab binding site**		None		
	**Palivizumab binding site**		None		
		**MELODY full cohort – RSV B**			
**Through 150 days post-dose**			**n = 12**	**n = 2**	**n = 14**
	F12I	0.07	3 (25)	0 (0)	3 (21.4)
	F15L	99.57	12 (100)	2 (100)	14 (100)
	A16V	1.57	1 (8.3)	0 (0)	1 (7.1)
	N18S	0.21	1 (8.3)	0 (0)	1 (7.1)
	L22P	1.68	1 (8.3)	0 (0)	1 (7.1)
	A103V	98.5	12 (100)	2 (100)	14 (100)
	A111V	0.07	1 (8.3)	0 (0)	1 (7.1)
	L172Q	98.61	12 (100)	2 (100)	14 (100)
	S173L	98.36	11 (91.7)	1 (50)	12 (85.7)
	S190N	2.79	10 (83.3)	1 (50)	11 (78.6)
	K191R	68.61	11 (91.7)	2 (100)	13 (92.9)
	I206M	68.89	11 (91.7)	2 (100)	13 (92.9)
	Q209R	68.18	11 (91.7)	2 (100)	13 (92.9)
	S211N	1.14	10 (83.3)	1 (50)	11 (78.6)
	V239I	0.14	1 (8.3)	0 (0)	1 (7.1)
	K327E	0.04	1 (8.3)	0 (0)	1 (7.1)

	S389P	1.07	10 (83.3)	1 (50)	11 (78.6)
	S436P	NA	1 (8.3)	0 (0)	1 (7.1)
	T522A	0.07	1 (8.3)	0 (0)	1 (7.1)
	A529V	0.93	1 (8.3)	0 (0)	1 (7.1)
	**Nirsevimab binding site**				
	I206M	68.89	11 (91.7)	2 (100)	13 (92.9)
	Q209R	68.18	11 (91.7)	2 (100)	13 (92.9)
	S211N	1.14	10 (83.3)	1 (50)	11 (78.6)
	**Palivizumab binding site**		None		
**151–360 days post-dose**	**Nirsevimab binding site**		None		
	**Palivizumab binding site**		None		
**361–511 days post-dose**	**Nirsevimab binding site**		None		
	**Palivizumab binding site**		None		

Major variants had ≥25% MAF in surveillance studies. Site Ø=AAs 62–96 and 195–227; Site II=AAs 254–277; non-EC regions: signal peptide=AAs 1–23; p27=AAs 110–136; transmembrane and intracellular domains=AAs 525–574; nirsevimab binding site=AAs 62–69 and 196–212; palivizumab binding site=AAs 262–275.

AA, amino acid; EC, extracellular; MAF, molecular allele frequency; NA, not applicable (i.e. not observed); RSV, respiratory syncytial virus.

Supplementary Table S4 | Prevalence and frequency of all major variant substitutions observed for cases that met the exploratory case definition of RSV unscheduled event in the Phase 2b and MELODY trials.

Sampling period	Amino acid substitutions in RSV F	Prevalence in surveillance studies (%)	Frequency		
			Placebo n (%)	Nirsevimab n (%)	Total n (%)
		Phase 2b – RSV A			
**Through 150 days post-dose**			**n = 0**	**n = 3**	**n = 3**
	T13A	4.66	0 (0)	1 (33.3)	1 (33.3)
	L119I	0.8	0 (0)	2 (66.7)	2 (66.7)
	K209R	NA	0 (0)	2 (66.7)	2 (66.7)
	**Nirsevimab binding site**				
	K209R	NA	0 (0)	2 (66.7)	2 (66.7)
	**Palivizumab binding site**		None		
**151–360 days post-dose**	**Nirsevimab binding site**		None		
	**Palivizumab binding site**		None		
		Phase 2b – RSV B			
**Through 150 days post-dose**			**n = 0**	**n = 1**	**n = 1**
	F15L	99.57	0 (0)	1 (100)	1 (100)
	A103V	98.5	0 (0)	1 (100)	1 (100)
	I129M	0.43	0 (0)	1 (100)	1 (100)
	L172Q	98.61	0 (0)	1 (100)	1 (100)
	S173L	98.36	0 (0)	1 (100)	1 (100)
	K191R	68.61	0 (0)	1 (100)	1 (100)
	I206M	68.89	0 (0)	1 (100)	1 (100)
	Q209R	68.18	0 (0)	1 (100)	1 (100)
	T518I	NA	0 (0)	1 (100)	1 (100)

	**Nirsevimab binding site**				
	I206M	68.89	0 (0)	1 (100)	1 (100)
	Q209R	68.18	0 (0)	1 (100)	1 (100)
	**Palivizumab binding site**		None		
**151–360 days post-dose**			**n = 1**	**n = 0**	**n = 1**
	F12L	7.21	1 (100)	0 (0)	1 (100)
	F15L	99.57	1 (100)	0 (0)	1 (100)
	A103V	98.5	1 (100)	0 (0)	1 (100)
	L172Q	98.61	1 (100)	0 (0)	1 (100)
	S173L	98.36	1 (100)	0 (0)	1 (100)
	S190N	2.79	1 (100)	0 (0)	1 (100)
	K191R	68.61	1 (100)	0 (0)	1 (100)
	I206M	68.89	1 (100)	0 (0)	1 (100)
	Q209R	68.18	1 (100)	0 (0)	1 (100)
	S276N	7.14	1 (100)	0 (0)	1 (100)
	E463D	7	1 (100)	0 (0)	1 (100)
	**Nirsevimab binding site**				
	I206M	68.89	1 (100)	0 (0)	1 (100)
	Q209R	68.18	1 (100)	0 (0)	1 (100)
	**Palivizumab binding site**		None		
		**MELODY full cohort – RSV A**			
**Through 150 days post-dose**			**n = 7**	**n = 5**	**n = 12**
	T12I	6.75	1 (14.3)	1 (20)	2 (16.7)
	T13A	4.66	2 (28.6)	0 (0)	2 (16.7)
	L15F	2.09	1 (14.3)	0 (0)	1 (8.3)
	A103T	1.91	1 (14.3)	1 (20)	2 (16.7)

	T122A	11.58	2 (28.6)	1 (20)	3 (25)
	S276N	2.05	0 (0)	1 (20)	1 (8.3)
	N325Y		1 (14.3)	0 (0)	1 (8.3)
	S330T	0.03	1 (14.3)	0 (0)	1 (8.3)
	S362L	0.97	1 (14.3)	0 (0)	1 (8.3)
	K419N	0.03	1 (14.3)	0 (0)	1 (8.3)
	A518V	0.7	0 (0)	1 (20)	1 (8.3)
	A540V		0 (0)	1 (20)	1 (8.3)
	A552T	0.14	1 (14.3)	0 (0)	1 (8.3)
	**Nirsevimab binding site**		None		
	**Palivizumab binding site**		None		
**151–360 days post-dose**			**n = 1**	**n = 1**	**n = 2**
	L111I	0.14	0 (0)	1 (100)	1 (50)
	T122A	11.58	1 (100)	1 (100)	2 (100)
	K123Q	3.62	1 (100)	0 (0)	1 (50)
	E378D	0.03	0 (0)	1 (100)	1 (50)
	I384T	3.76	1 (100)	0 (0)	1 (50)
	**Nirsevimab binding site**		None		
	**Palivizumab binding site**		None		
**361–511 days post-dose**			**n = 0**	**n = 1**	**n = 1**
	T12I	6.75	0 (0)	1 (100)	1 (100)
	S105N	0.45	0 (0	1 (100)	1 (100)
	E497D	NA	0 (0)	1 (100)	1 (100)
	**Nirsevimab binding site**		None		
	**Palivizumab binding site**		None		

		MELODY full cohort – RSV B			
**Through 150 days post-dose**			**n = 2**	**n = 1**	**n = 3**
	F12I	0.07	1 (50)	0 (0)	1 (33.3)
	F15L	99.57	2 (100)	1 (100)	3 (100)
	A19T	2.25	0 (0)	1 (100)	1 (33.3)
	N99T	NA	1 (50)	0 (0)	1 (33.3)
	A103V	98.5	2 (100)	1 (100)	3 (100)
	L172Q	98.61	2 (100)	1 (100)	3 (100)
	S173L	98.36	1 (50)	1 (100)	2 (66.7)
	S190N	2.79	2 (100)	1 (100)	3 (100)
	K191R	68.61	2 (100)	1 (100)	3 (100)
	I206M	68.89	2 (100)	1 (100)	3 (100)
	Q209R	68.18	2 (100)	1 (100)	3 (100)
	S211N	1.14	2 (100)	1 (100)	3 (100)
	S389P	1.07	2 (100)	1 (100)	3 (100)
	**Nirsevimab binding site**		None		
	I206M	68.89	2 (100)	1 (100)	3 (100)
	Q209R	68.18	2 (100)	1 (100)	3 (100)
	S211N	1.14	2 (100)	1 (100)	3 (100)
	**Palivizumab binding site**		None		

Major variants had ≥25% MAF in surveillance studies. Site Ø=AAs 62–96 and 195–227; Site II=AAs 254–277; non-EC regions: signal peptide=AAs 1–23; p27=AAs 110–136; transmembrane and intracellular domains=AAs 525–574; nirsevimab binding site=AAs 62–69 and 196–212; palivizumab binding site=AAs 262–275.

AA, amino acid; EC, extracellular; MAF, molecular allele frequency; NA, not applicable (i.e. not observed); RSV, respiratory syncytial virus.

Supplementary Table S5 | Prevalence and frequency of all major variant substitutions observed for cases that met the exploratory case definition of non-protocol defined LRTI outpatient event in the Phase 2b and MELODY trials.

Sampling period	Amino acid substitutions in RSV F	Prevalence in surveillance studies (%)	Frequency		
			**Placebo n (%)**	**Nirsevimab n (%)**	**Total n (%)**
		**Phase 2b – RSV A**			
**Through 150 days post-dose**			**n = 1**	**n = 2**	**n = 3**
	A103V	0.28	0 (0)	1 (50)	1 (33.3)
	G329R	0.07	1 (100)	1 (50)	2 (66.7)
	**Nirsevimab binding site**		None		
	**Palivizumab binding site**		None		
		**Phase 2b – RSV B**			
**Through 150 days post-dose**			**n = 2**	**n = 2**	**n = 4**
	F15L	99.57	2 (100)	2 (100)	4 (100)
	A103V	98.5	2 (100)	2 (100)	4 (100)
	L172Q	98.61	2 (100)	2 (100)	4 (100)
	S173L	98.36	2 (100)	2 (100)	4 (100)
	K191R	68.61	2 (100)	1 (50)	3 (75)
	I206M	68.89	2 (100)	1 (50)	3 (75)
	Q209R	68.18	2 (100)	1 (50)	3 (75)
	V365A	0.07	1 (50)	1 (50)	2 (50)
	**Nirsevimab binding site**		None		
	I206M	68.89	2 (100)	1 (50)	3 (75)
	Q209R	68.18	2 (100)	1 (50)	3 (75)
	**Palivizumab binding site**		None		

**MELODY full cohort RSV A**					
**Through 150 days post-dose**			**n = 7**	**n = 5**	**n = 12**
	T12I	6.75	1 (14.3)	1 (20)	2 (16.7)
	L15F	2.09	0 (0)	1 (20)	1 (8.3)
	S24F	0.45	0 (0)	1 (20)	1 (8.3)
	A103T	1.91	1 (14.3)	0 (0)	1 (8.3)
	N116S	0.1	1 (14.3)	0 (0)	1 (8.3)
	T122A	11.58	0 (0)	2 (40)	2 (16.7)
	T125A	0.21	0 (0)	1 (20)	1 (8.3)
	S276N	2.05	0 (0)	1 (20)	1 (8.3)
	A355V	0.24	0 (0)	1 (20)	1 (8.3)
	**Nirsevimab binding site**		None		
	**Palivizumab binding site**		None		
**151–360 days post-dose**			**n = 2**	**n = 2**	**n = 4**
	T12I	6.75	1 (50)	0 (0)	1 (25)
	L20F	0.77	1 (50)	0 (0)	1 (25)
	I57V	NA	0 (0)	1 (50)	1 (25)
	L111I	0.14	0 (0)	1 (50)	1 (25)
	T122A	11.58	1 (50)	2 (100)	3 (75)
	K123Q	3.62	1 (50)	1 (50)	2 (50)
	I384T	3.76	1 (50)	1 (50)	2 (50)
	**Nirsevimab binding site**		None		
	**Palivizumab binding site**		None		
**361–511 days post-dose**			**n = 1**	**n = 0**	**n = 1**
	T122A	11.58	1 (100)	0 (0)	1 (100)
	K123Q	3.62	1 (100)	0 (0)	1 (100)

	I384T	3.76	1 (100)	0 (0)	1 (100)
	**Nirsevimab binding site**		None		
	**Palivizumab binding site**		None		
		**MELODY full cohort RSV B**			
**Through 150 days post-dose**			**n = 5**	**n = 0**	**n = 5**
	F12I	0.07	2 (40)	0 (0)	2 (40)
	F15L	99.57	5 (100)	0 (0)	5 (100)
	A103V	98.5	5 (100)	0 (0)	5 (100)
	L172Q	98.61	5 (100)	0 (0)	5 (100)
	S173L	98.36	5 (100)	0 (0)	5 (100)
	S190N	2.79	4 (80)	0 (0)	4 (80)
	K191R	68.61	5 (100)	0 (0)	5 (100)
	I206M	68.89	5 (100)	0 (0)	5 (100)
	Q209R	68.18	5 (100)	0 (0)	5 (100)
	S211N	1.14	4 (80)	0 (0)	4 (80)
	S330A	NA	1 (20)	0 (0)	1 (20)
	S389P	1.07	4 (80)	0 (0)	4 (80)
	N437I	NA	1 (20)	0 (0)	1 (20)
	**Nirsevimab binding site**				
	I206M	68.89	5 (100)	0 (0)	5 (100)
	Q209R	68.18	5 (100)	0 (0)	5 (100)
	S211N	1.14	4 (80)	0 (0)	4 (80)
	**Palivizumab binding site**		None		

Major variants had ≥25% MAF in surveillance studies. Site Ø=AAs 62–96 and 195–227; Site II=AAs 254–277; non-EC regions: signal peptide=AAs 1–23; p27=AAs 110–136; transmembrane and intracellular domains=AAs 525–574; nirsevimab binding site=AAs 62–69 and 196–212; palivizumab binding site=AAs 262–275.

AA, amino acid; EC, extracellular; MAF, molecular allele frequency; NA, not applicable (i.e. not observed); RSV, respiratory syncytial virus.

Supplementary Table S6 | Prevalence and frequency of all major variant substitutions observed for cases that met the exploratory case definition of hospitalization due to RSV non-LRTI in the Phase 2b and MELODY trials.

Sampling period	Amino acid substitutions in RSV F	Prevalence in surveillance studies (%)	Frequency		
			Placebo n (%)	Nirsevimab n (%)	Total n (%)
		**Phase 2b – RSV A**			
**Through 150 days post-dose**	**Nirsevimab binding site**		None		
	**Palivizumab binding site**		None		
**151–360 days post-dose**	**Nirsevimab binding site**		None		
	**Palivizumab binding site**		None		
		**Phase 2b – RSV B**			
**Through 150 days post-dose**	**Nirsevimab binding site**		None		
	**Palivizumab Binding Site**		None		
**151–360 days post-dose**	**Nirsevimab binding site**		None		
	**Palivizumab binding site**		None		
		**MELODY full cohort – RSV A**			
**Through 150 days post-dose**			**n = 0**	**n = 1**	**n = 1**
	T12I	6.75	0 (0)	1 (100)	1 (100)
	S330T	0.03	0 (0)	1 (100)	1 (100)
	V360A	NA	0 (0)	1 (100)	1 (100)
	A552T	0.14	0 (0)	1 (100)	1 (100)
	**Nirsevimab binding site**		None		
	**Palivizumab binding site**		None		
**151–360 days post-dose**	**Nirsevimab binding site**		None		
	**Palivizumab binding site**		None		
		**MELODY full cohort – RSV B**			

**Through 150 days post-dose**	**Nirsevimab binding site**	None
	**Palivizumab binding site**	None
**151–360 days post-dose**	**Nirsevimab binding site**	None
	**Palivizumab binding site**	None

Major variants had ≥25% MAF in surveillance studies. Site Ø=AAs 62–96 and 195–227; Site II=AAs 254–277; non-EC regions: signal peptide=AAs 1–23; p27=AAs 110–136; transmembrane and intracellular domains=AAs 525–574; nirsevimab binding site=AAs 62–69 and 196–212; palivizumab binding site=AAs 262–275.

AA, amino acid; EC, extracellular; IC_{50}, half maximal inhibitory concentration; MAF, molecular allele frequency; NA, not applicable (i.e. not observed); RSV, respiratory syncytial virus.

Supplementary Table S7 | Neutralization potency of nirsevimab and palivizumab on all major variant substitutions in the extracellular domain of the RSV A and RSV B F protein observed for cases that met the primary case definition in the Phase 2b and MELODY trials.

Sampling period	Amino acid substitutions	Fold change in IC_{50}	
		**Nirsevimab**	**Palivizumab**
	**Phase 2b – RSV A**		
**Through 150 days post-dose**	S25N	2.47	1.94
	G71S	QNS	QNS
	V76I	1.23	0.88
	S99N	5.25	2.11
	A102S	1.41	0.94
	S213R	2.24	2.57
	K419E	QNS	QNS
	**Nirsevimab binding site**	None	
	**Palivizumab binding site**	None	
**151–360 days post-dose**	S190N	2.17	2.62
	**Nirsevimab binding site**	None	
	**Palivizumab binding site**	None	
	**Phase 2b – RSV B**		
**Through 150 days post-dose**	I64T	>496.28	5.19
	K68E	>283.42	2.10
	A103V	0.89	1.76
	L172Q	1.12	2.26
	S173L	0.88	1.88
	K191R	1.26	2.73
	I206M	5.02	1.96
	N208S	>386.60	1.81

	Q209R	0.47	3.12
	S276N	2.04	2.55
	K327R	0.89	1.86
	E463D	1.39	2.12
	**Nirsevimab binding site**		
	I64T	>496.28	5.19
	K68E	>283.42	2.10
	I206M	5.02	1.96
	N208S	>386.60	1.81
	Q209R	0.47	3.12
	**Palivizumab binding site**	None	
**151–360 days post-dose**	Y33F	0.49	1.20
	A103V	0.89	1.76
	L172Q	1.12	2.26
	S173L	0.88	1.88
	K191R	1.26	2.73
	I206M	5.02	1.96
	Q209R	0.47	3.12
	**Nirsevimab binding site**		
	I206M	5.02	1.96
	Q209R	0.47	3.12
	**Palivizumab binding site**	None	
	**MELODY full cohort – RSV A**		
**Through 150 days post-dose**	A103T	1.0	1.0
	A107T	2.13	2.30
	V144I	1.14	1.13

	T245N	NA	NA
	S255N	NA	NA
	S276N	1.0	1.0
	Q354R	2.12	1.93
	I384T	2.40	1.35
	V406I	NA	NA
	K419E	QNS	QNS
	D479N	NA	NA
	D486N	NA	NA
	**Nirsevimab binding site**	None	
	**Palivizumab binding site**	None	
**151–360 days post-dose**	I384T	2.40	1.35
	**Nirsevimab binding site**	None	
	**Palivizumab binding site**	None	
**361–511 days post-dose**	E378D	2.25	1.36
	I384T	2.40	1.35
	S443T	NA	NA
	**Nirsevimab binding site**	None	
	**Palivizumab binding site**	None	
	**MELODY full cohort – RSV B**		
**Through 150 days post-dose**	T91N	NA	NA
	A103V	0.89	1.76
	L172Q	1.12	2.26
	S173L	0.88	1.88
	S190N	QNS	QNS
	K191R	1.26	2.73

	L204S	NA	NA
	I206M	5.02	1.96
	Q209R	0.47	3.12
	S211N	1.24	1.86
	V239I	NA	NA
	K272R	NA	41.80
	K327E	NA	1.74
	V365I	NA	NA
	P376S	NA	NA
	S389P	NA	NA
	S436P	NA	NA
	T522A	NA	NA
	**Nirsevimab binding site**		
	L204S	NA	NA
	I206M	5.02	1.96
	Q209R	0.47	3.12
	S211N	1.24	1.86
	**Palivizumab binding site**		
	K272R	NA	41.80
**151–360 days post-dose**	A103V	0.89	1.76
	L172Q	1.12	2.26
	S173L	0.88	1.88
	S190N	QNS	QNS
	K191R	1.26	2.73
	I206M	5.02	1.96
	Q209R	0.47	3.12

	S211N	1.24	1.86
	S330T	0.81	1.89
	S389P	NA	NA
	**Nirsevimab binding site**		
	I206M	5.02	1.96
	Q209R	0.47	3.12
	S211N	1.24	1.86
	**Palivizumab binding site**	None	
**361–511 days post-dose**	A103V	0.89	1.76
	L172Q	1.12	2.26
	S173L	0.88	1.88
	K191R	1.26	2.73
	I206M	5.02	1.96
	Q209R	0.47	3.12
	S330T	0.81	1.89
	**Nirsevimab binding site**		
	I206M	5.02	1.96
	Q209R	0.47	3.12
	**Palivizumab binding site**	None	

Major variants had ≥25% MAF in surveillance studies. Site Ø=AAs 62–96 and 195–227; Site II=AAs 254–277; non-EC regions: signal peptide=AAs 1–23; p27=AAs 110–136; transmembrane and intracellular domains=AAs 525–574; nirsevimab binding site=AAs 62–69 and 196–212; palivizumab binding site=AAs 262–275.

AA, amino acid; EC, extracellular; IC_{50}, half maximal inhibitory concentration; MAF, molecular allele frequency; NA, not applicable (i.e. not observed); QNS, quantity not sufficient; RSV, respiratory syncytial virus.

Supplementary Table S8 | Neutralization potency of nirsevimab and palivizumab on all minor variant substitutions in the extracellular domain of the RSV A and RSV B F protein observed for cases that met the primary case definition in the Phase 2b and MELODY trials.

Sampling period	Amino acid substitutions in RSV F	Fold change in IC_{50}	
		**Nirsevimab**	**Palivizumab**
	**Phase 2b – RSV A**		
**Through 150 days post-dose**	R49K	NA	NA
	**Nirsevimab binding site**	None	
	**Palivizumab binding site**	None	
	**Phase 2b – RSV B**		
**Through 150 days post-dose**	L171M	NA	NA
	N208K	>350.47	2.05
	L381F	NA	NA
	Y457H	QNS	QNS
	**Nirsevimab binding site**		
	N208K	>350.47	2.05
	**Palivizumab binding site**	None	
	**MELODY full cohort – RSV A**		
**Through day 150 post-dose**	S105N	1.0	1.0
	N165S	NA	NA
	N228S	NA	NA
	V247L	NA	NA
	S276N	1.0	1.0
	N515H	NA	NA
	**Nirsevimab binding site**	None	
	**Palivizumab binding site**	None	

**151–360 days post-dose**	K272E	NA	41.80
	W341R	NA	NA
	A355V	NA	NA
	I384V	1.0	1.0
	N515H	NA	NA
	**Nirsevimab binding site**	None	
	**Palivizumab binding site**	None	
	K272E	NA	41.80
	K42R	NA	NA
**361–511 days post-dose**	N67T	NA	NA
	A74T	NA	NA
	L78F	NA	NA
	S169N	NA	NA
	D200N	NA	NA
	K201N	NA	NA
	S213R	2.24	2.57
	N228S	NA	NA
	V247L	NA	NA
	**Nirsevimab binding site**		
	N67T	NA	NA
	D200N	NA	NA
	K201N	NA	NA
	**Palivizumab binding site**	None	
	**MELODY full cohort – RSV B**		
**Through 150 days post-dose**	I64T	>496.28	5.19
	K65E	NA	NA

	K68E	>283.42	2.10
	N200Y	NA	NA
	N208I	NA	NA
	I475M	NA	NA
	Y477H	NA	NA
	**Nirsevimab binding site**		
	I64T	>496.28	5.19
	K65E	NA	NA
	K68E	>283.42	2.10
	N200Y	NA	NA
	N208I	NA	NA
	**Palivizumab binding site**	None	
**151–360 days post-dose**	K87R	NA	NA
	**Nirsevimab binding site**	None	
	**Palivizumab binding site**	None	
**361–511 days post-dose**	**Nirsevimab binding site**	None	
	**Palivizumab binding site**	None	

Minor variants had RSV A ≥4%–<25% MAF; RSV B ≥5%–<25% MAF in surveillance studies. Site Ø=AAs 62–96 and 195–227; Site II=AAs 254–277; non-EC regions: signal peptide=AAs 1–23; p27=AAs 110–136; transmembrane and intracellular domains=AAs 525–574; nirsevimab binding site=AAs 62–69 and 196–212; palivizumab binding site=AAs 262–275.

AA, amino acid; EC, extracellular; IC_{50}, half maximal inhibitory concentration; MAF, molecular allele frequency; NA, not applicable (i.e., not observed); QNS, quantity not sufficient; RSV, respiratory syncytial virus.

Supplementary Table S9 | Neutralization potency of nirsevimab and palivizumab on all major variant substitutions in the extracellular domain of the RSV A and RSV B F protein observed for cases that met the secondary case definition in the Phase 2b and MELODY trials.

Sampling period	Amino acid substitutions in RSV F	Fold change in IC_{50}	
		Nirsevimab	Palivizumab
	**Phase 2b – RSV A**		
**Through 150 days post-dose**	A102S	1.41	0.94
	S213R	2.24	2.57
	K419E	QNS	QNS
	**Nirsevimab binding site**	None	
	**Palivizumab binding site**	None	
**151–360 days post-dose**	**Nirsevimab binding site**	None	
	**Palivizumab binding site**	None	
	**Phase 2b – RSV B**		
**Through 150 days post-dose**	I64T	>496.28	5.19
	K68E	>283.42	2.10
	A103V	0.89	1.76
	L172Q	1.12	2.26
	S173L	0.88	1.88
	K191R	1.26	2.73
	I206M	5.02	1.96
	N208S	>386.60	1.81
	Q209R	0.47	3.12
	S276N	2.04	2.55
	K327R	0.89	1.86
	E463D	1.39	2.12
	**Nirsevimab binding site**	None	

	I64T	>496.28	5.19
	K68E	>283.42	2.10
	I206M	5.02	1.96
	N208S	>386.60	1.81
	Q209R	0.47	3.12
	**Palivizumab binding site**	None	
**151–360 days post-dose**	A103V	0.89	1.76
	L172Q	1.12	2.26
	S173L	0.88	1.88
	K191R	1.26	2.73
	I206M	5.02	1.96
	Q209R	0.47	3.12
	**Nirsevimab binding site**	None	
	I206M	5.02	1.96
	Q209R	0.47	3.12
	**Palivizumab binding site**	None	
**MELODY full cohort – RSV A**			
**Through 150 days post-dose**	A103T	1.0	1.0
	A107T	2.13	2.30
	S276N	1.0	1.0
	K419E	QNS	QNS
	D486N	NA	NA
	**Nirsevimab binding site**	None	
	**Palivizumab binding site**	None	
**151–360 days post-dose**	I384T	2.40	1.35
	**Nirsevimab binding site**	None	

	**Palivizumab binding site**	None	
**361–511 days post-dose**	**Nirsevimab binding site**	None	
	**Palivizumab binding site**	None	
	**MELODY full cohort – RSV B**		
**Through 150 days post-dose**	A103V	0.89	1.76
	L172Q	1.12	2.26
	S173L	0.88	1.88
	S190N	NA	NA
	K191R	1.26	2.73
	I206M	5.02	1.96
	Q209R	0.47	3.12
	S211N	1.24	1.86
	V239I	NA	NA
	K327E	NA	1.74
	S389P	NA	NA
	S436P	NA	NA
	T522A	NA	NA
	**Nirsevimab binding site**		
	I206M	5.02	1.96
	Q209R	0.47	3.12
	S211N	1.24	1.86
	**Palivizumab binding site**	None	
**151–360 days post-dose**	**Nirsevimab binding site**	None	
	**Palivizumab binding site**	None	
**361–511 days post-dose**	**Nirsevimab binding site**	None	
	**Palivizumab binding site**	None	

Major variants had ≥25% MAF in surveillance studies. Site Ø=AAs 62–96 and 195–227; Site II=AAs 254–277; non-EC regions: signal peptide=AAs 1–23; p27=AAs 110–136; transmembrane and intracellular domains=AAs 525–574; nirsevimab binding site=AAs 62–69 and 196–212; palivizumab binding site=AAs 262–275.

AA, amino acid; EC, extracellular; IC_{50}, half maximal inhibitory concentration; NA, not applicable (i.e. not observed); QNS, quantity not sufficient; RSV, respiratory syncytial virus.

Supplementary Table S10 | Neutralization potency of nirsevimab and palivizumab on all major variant substitutions in the extracellular domain of the RSV A and RSV B F protein observed for cases that met the exploratory case definition of RSV unscheduled event in the Phase 2b and MELODY trials.

Sampling period	Amino acid substitutions in RSV F	Fold change in IC_{50}	
		**Nirsevimab**	**Palivizumab**
	**Phase 2b – RSV A**		
**Through 150 days post-dose**	K209R	0.93	1.01
	**Nirsevimab binding site**		
	K209R	0.93	1.01
	**Palivizumab binding site**	None	
**151–360 days post-dose**	**Nirsevimab binding site**	None	
	**Palivizumab binding site**	None	
	**Phase 2b – RSV B**		
**Through 150 days post-dose**	A103V	0.89	1.76
	L172Q	1.12	2.26
	S173L	0.88	1.88
	K191R	1.26	2.73
	I206M	5.02	1.96
	Q209R	0.47	3.12
	T518I	2.97	4.39
	**Nirsevimab binding site**		
	I206M	5.02	1.96
	Q209R	0.47	3.12
	**Palivizumab binding site**	None	
**151–360 days post-dose**	A103V	0.89	1.76
	L172Q	1.12	2.26
	S173L	0.88	1.88

	S190N	QNS	QNS
	K191R	1.26	2.73
	I206M	5.02	1.96
	Q209R	0.47	3.12
	S276N	2.04	2.55
	E463D	1.39	2.12
	**Nirsevimab binding site**		
	I206M	5.02	1.96
	Q209R	0.47	3.12
	**Palivizumab binding site**	None	
	**MELODY full cohort – RSV A**		
**Through 150 days post-dose**	A103T	1.0	1.0
	S276N	1.0	1.0
	N325Y	1.83	1.53
	S330T	NA	1.07
	S362L	NA	NA
	K419N	NA	NA
	A518V	0.98	1.24
	**Nirsevimab binding site**	None	
	**Palivizumab binding site**	None	
**151–360 days post-dose**	E378D	2.25	1.36
	I384T	2.40	1.35
	**Nirsevimab binding site**	None	
	**Palivizumab binding site**	None	
**361–511 days post-dose**	S105N	1.0	1.0
	E497D	NA	NA

	**Nirsevimab binding site**	None	
	**Palivizumab binding site**	None	
	**MELODY full cohort – RSV B**		
**Through 150 days post-dose**	N99T	NA	NA
	A103V	0.89	1.76
	L172Q	1.12	2.26
	S173L	0.88	1.88
	S190N	NA	NA
	K191R	1.26	2.73
	I206M	5.02	1.96
	Q209R	0.47	3.12
	S211N	1.24	1.86
	S389P	NA	NA
	**Nirsevimab binding site**	None	
	I206M	5.02	1.96
	Q209R	0.47	3.12
	S211N	1.24	1.86
	**Palivizumab binding site**	None	
**151-360 days post-dose**	**Nirsevimab binding site**	None	
	**Palivizumab binding site**	None	
**361-511 days post-dose**	**Nirsevimab binding site**	None	
	**Palivizumab binding site**	None	

Major variants had ≥25% MAF in surveillance studies. Site Ø=AAs 62–96 and 195–227; Site II=AAs 254–277; non-EC regions: signal peptide=AAs 1–23; p27=AAs 110–136; transmembrane and intracellular domains=AAs 525–574; nirsevimab binding site=AAs 62–69 and 196–212; palivizumab binding site=AAs 262–275.

AA, amino acid; EC, extracellular; IC_{50}, half maximal inhibitory concentration; MAF, molecular allele frequency; NA, not applicable (i.e. not observed); QNS, quality not sufficient; RSV, respiratory syncytial virus.

Supplementary Table S11 | Neutralization potency of nirsevimab and palivizumab on all major variant substitutions in the extracellular domain of the RSV A and RSV B F protein observed for cases that met the exploratory case definition of non-protocol defined LRTI outpatient event in the Phase 2b and MELODY trials.

Sampling period	Amino acid substitutions in RSV F	Fold change in IC_{50}	
		Nirsevimab	Palivizumab
	**Phase 2b – RSV A**		
**Through 150 days post-dose**	A103V	2.18	1.53
	G329R	2.06	1.32
	**Nirsevimab binding site**	None	
	**Palivizumab binding site**	None	
	**Phase 2b – RSV B**		
**Through 150 days post-dose**	A103V	0.89	1.76
	L172Q	1.12	2.26
	S173L	0.88	1.88
	K191R	1.26	2.73
	I206M	5.02	1.96
	Q209R	0.47	3.12
	V365A	0.79	0.95
	**Nirsevimab binding site**	None	
	I206M	5.02	1.96
	Q209R	0.47	3.12
	**Palivizumab binding site**	None	
	**MELODY full cohort – RSV A**		
**Through 150 days post-dose**	A103T	1.0	1.0
	S276N	1.0	1.0
	A355V	1.73	1.88

	**Nirsevimab binding site**	None	
	**Palivizumab binding site**	None	
**151–360 days post-dose**	I57V	NA	NA
	I384T	2.40	1.35
	**Nirsevimab binding site**	None	
	**Palivizumab binding site**	None	
**361–511 days post-dose**	I384T	2.40	1.35
	**Nirsevimab binding Site**	None	
	**Palivizumab binding site**	None	
	**MELODY full cohort – RSV B**		
**Through 150 days post-dose**	A103V	0.89	1.76
	L172Q	1.12	2.26
	S173L	0.88	1.88
	S190N	NA	NA
	K191R	1.26	2.73
	I206M	5.02	1.96
	Q209R	0.47	3.12
	S211N	1.24	1.86
	S330A	NA	NA
	S389P	NA	NA
	N437I	NA	NA
	**Nirsevimab binding site**		
	I206M	5.02	1.96
	Q209R	0.47	3.12
	S211N	1.24	1.86
	**Palivizumab binding site**	None	

Major variants had ≥25% MAF in surveillance studies. Site Ø=AAs 62–96 and 195–227; Site II=AAs 254–277; non-EC regions: signal peptide=AAs 1–23; p27=AAs 110–136; transmembrane and intracellular domains=AAs 525–574; nirsevimab binding site=AAs 62–69 and 196–212; palivizumab binding site=AAs 262–275.

AA, amino acid; EC, extracellular; IC_{50}, half maximal inhibitory concentration; MAF, molecular allele frequency; NA, not applicable (i.e. not observed); QNS: quantity not sufficient; RSV, respiratory syncytial virus.

Supplementary Table S12 | Neutralization potency of nirsevimab and palivizumab on all major variant substitutions in the extracellular domain of the RSV A and RSV B F protein observed for cases that met the exploratory case definition of hospitalization due to RSV non-LRTI in the Phase 2b and MELODY trials.

Sampling period	Amino acid substitutions in RSV F	Fold change in IC_{50}	
		**Nirsevimab**	**Palivizumab**
	**Phase 2b – RSV A**		
**Through 150 days post-dose**	**Nirsevimab binding site**	None	
	**Palivizumab binding site**	None	
	**Phase 2b – RSV B**		
**Through 150 days post-dose**	**Nirsevimab binding site**	None	
	**Palivizumab binding site**	None	
	**MELODY full cohort – RSV A**		
**Through 150 days post-dose**	S330T	NA	1.07
	V360A	NA	NA
	**Nirsevimab binding site**	None	
	**Palivizumab binding site**	None	
	**MELODY full cohort – RSV B**		
**Through 150 days post-dose**	**Nirsevimab binding site**	None	
	**Palivizumab binding site**	None	

Major variants had ≥25% MAF in surveillance studies. Site Ø=AAs 62–96 and 195–227; Site II=AAs 254–277; non-EC regions: signal peptide=AAs 1–23; p27=AAs 110–136; transmembrane and intracellular domains=AAs 525–574; nirsevimab binding site=AAs 62–69 and 196–212; palivizumab binding site=AAs 262–275.

AA, amino acid; EC, extracellular; IC_{50}, half maximal inhibitory concentration; MAF, molecular allele frequency; NA, not applicable (i.e. not observed); RSV, respiratory syncytial virus.

Supplementary Table S13 | Primer sequences used for qRT-PCR and genotyping.

Oligonucleotide	Sequence 5’ ---- 3'	Final concentration, nM
RSV A F1 forward	CRAAATHARMTCTGGGGCAAA	1600
RSV A F1 reverse	CCARCARGGWGTATCWATWACACCAT	1600
RSV A F2 forward	TCAATGATATGCCTATAACAAATGATC	800
RSV A F2 reverse	CAAGCAATGACCTCKAATYTC	800
RSV B F1 forward	CGAAATTAAATCTGGGGCAAA	600
RSV B F1 reverse	CCAGCAAGGTGTATCAATTACACCAT	600
RSV B F2 forward	TCAATGATATGCCTATAACAAATGATC	600
RSV B F2 reverse	CAAGCAATGACCTCTAATCTC	600

qRT-PCR, quantitative revere-transcriptase polymerase chain reaction; RSV, respiratory syncytial virus.

Supplementary Fig. S1 | CONSORT diagrams for A) Phase 2b and B) MELODY (full cohort) trials; any RSV infection.

A)

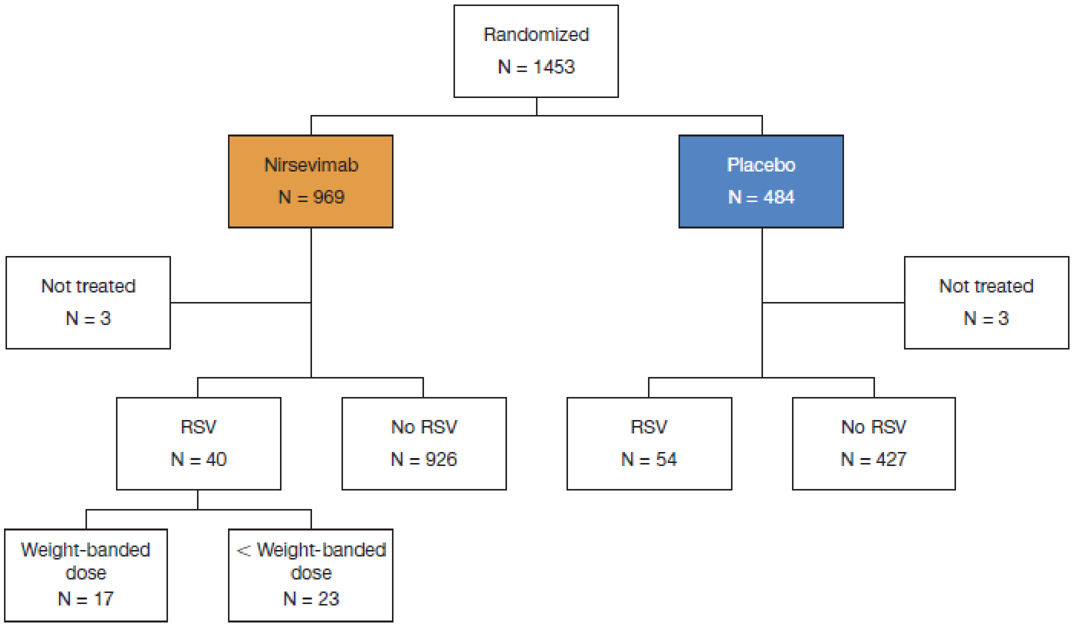

B)

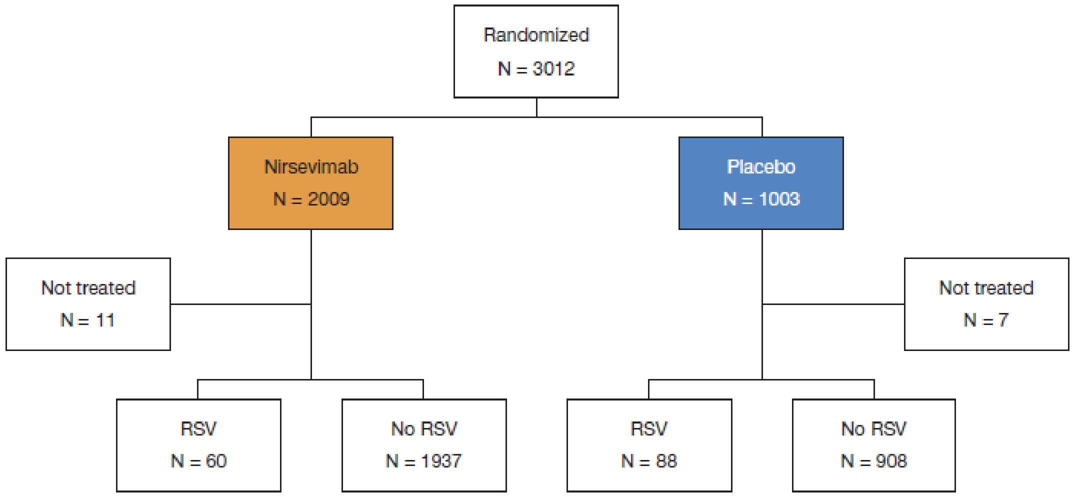

RSV cases shown are those with evaluable NGS data. For the Phase 2b trial, all infants received 50 mg, regardless of weight. For MELODY, nirsevimab was administered according to the weight-banded dosing regimen (participants <5 kg received 50 mg and those ≥5 kg received 100 mg nirsevimab).

NGS, next-generation sequencing; RSV, respiratory syncytial virus.

Supplementary Fig. S2 | Susceptibility of resistance-associated nirsevimab binding site substitutions N208S and K68E identified in Phase 2b and MELODY trials to neutralization by antibodies in serum from 17 healthy donors.

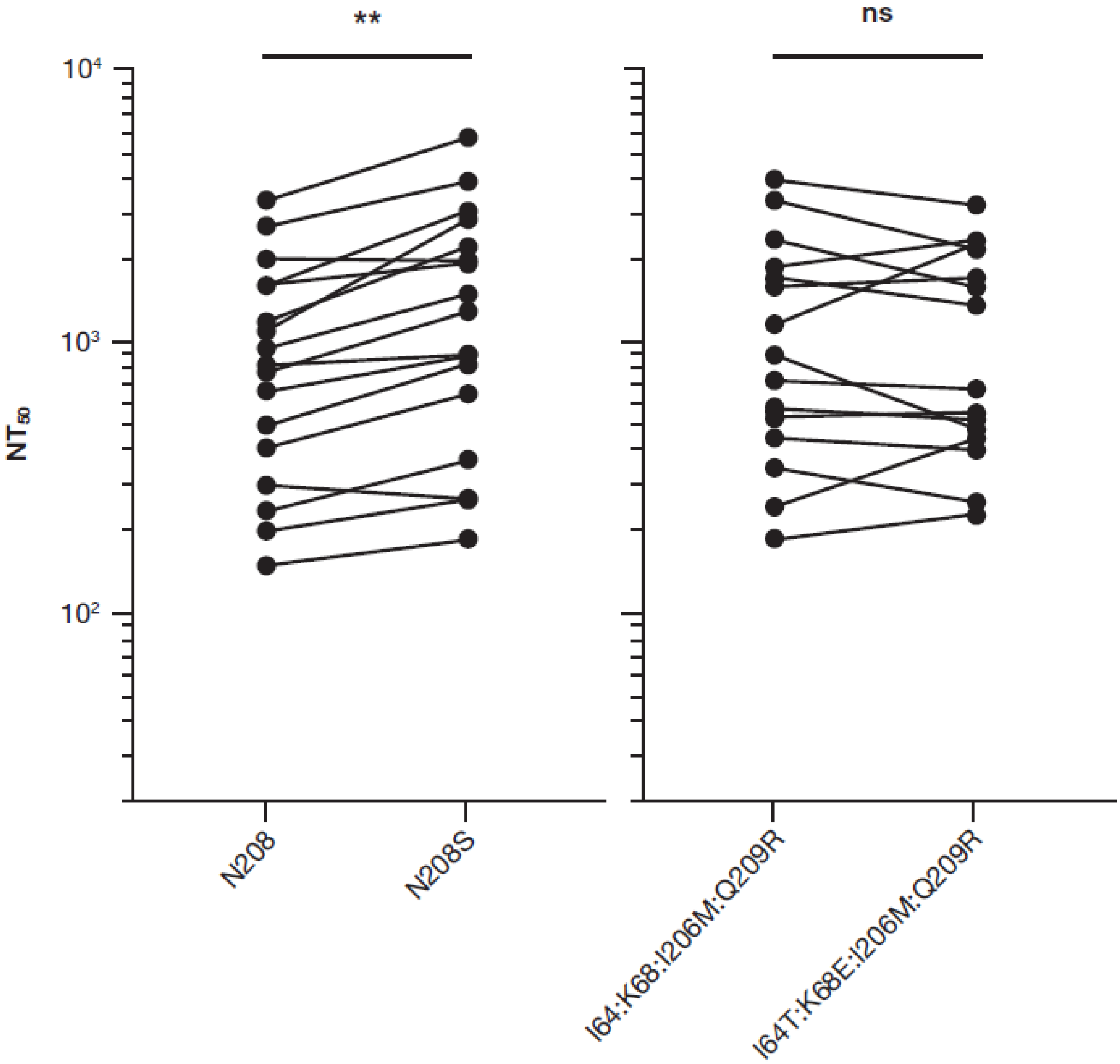

**p<0.05. N208S vs N208, p=0.0028; I64T:K68E:I206M:Q209R vs I64:K68:I206:Q209, p = 0.2639.

NT_{50} values were determined by fitting a four-parameter logistics model using GraphPad Prism 9.4.0. A paired statistical analysis of the corresponding recombinant viruses was performed using a two-tailed t-test. Multiple comparison analysis was not performed.

ns, non-significant; NT_{50}, half maximal neutralization titer.

Supplementary Fig. S3 | RSV viral titers in participants with resistance-associated substitutions in the Phase 2b and MELODY (primary cohort) studies, as determined by qRT-PCR.

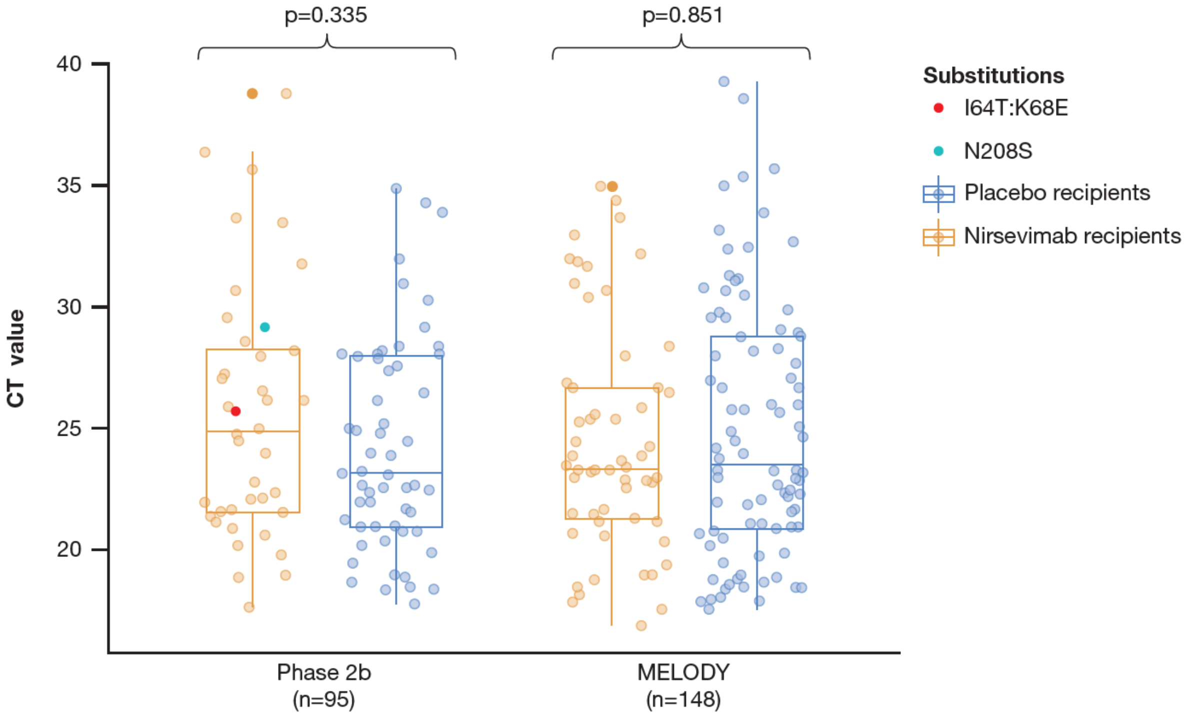

The blue and red dots represent CT values from individuals with N208S and I64T:K68E substitutions, respectively. Boxes indicate the 25th and 75th percentiles, the whiskers are 1.5x inter-quartile range. Filled yellow circles above the whiskers indicates where outliers for nirsevimab recipients fall. P-values were calculated using a two-sided Mann-Whitney test.

CT, cycle threshold; qRT-PCR, quantitative revere-transcriptase polymerase chain reaction; RSV, respiratory syncytial virus.

Supplementary Fig. S4 | RSV F protein site Ø conformational changes upon nirsevimab binding. (**A**) Structure and alignment of unbound F glycoprotein site Ø trimer from RSV B (dark green) in complex with nirsevimab (light gray), with largest conformational changes between unbound (dark gray) and bound states shown in angstroms (pink). (**B**) F glycoprotein RSV B monomers bound and unbound and (**C**) F glycoprotein RSV A monomers bound and unbound.

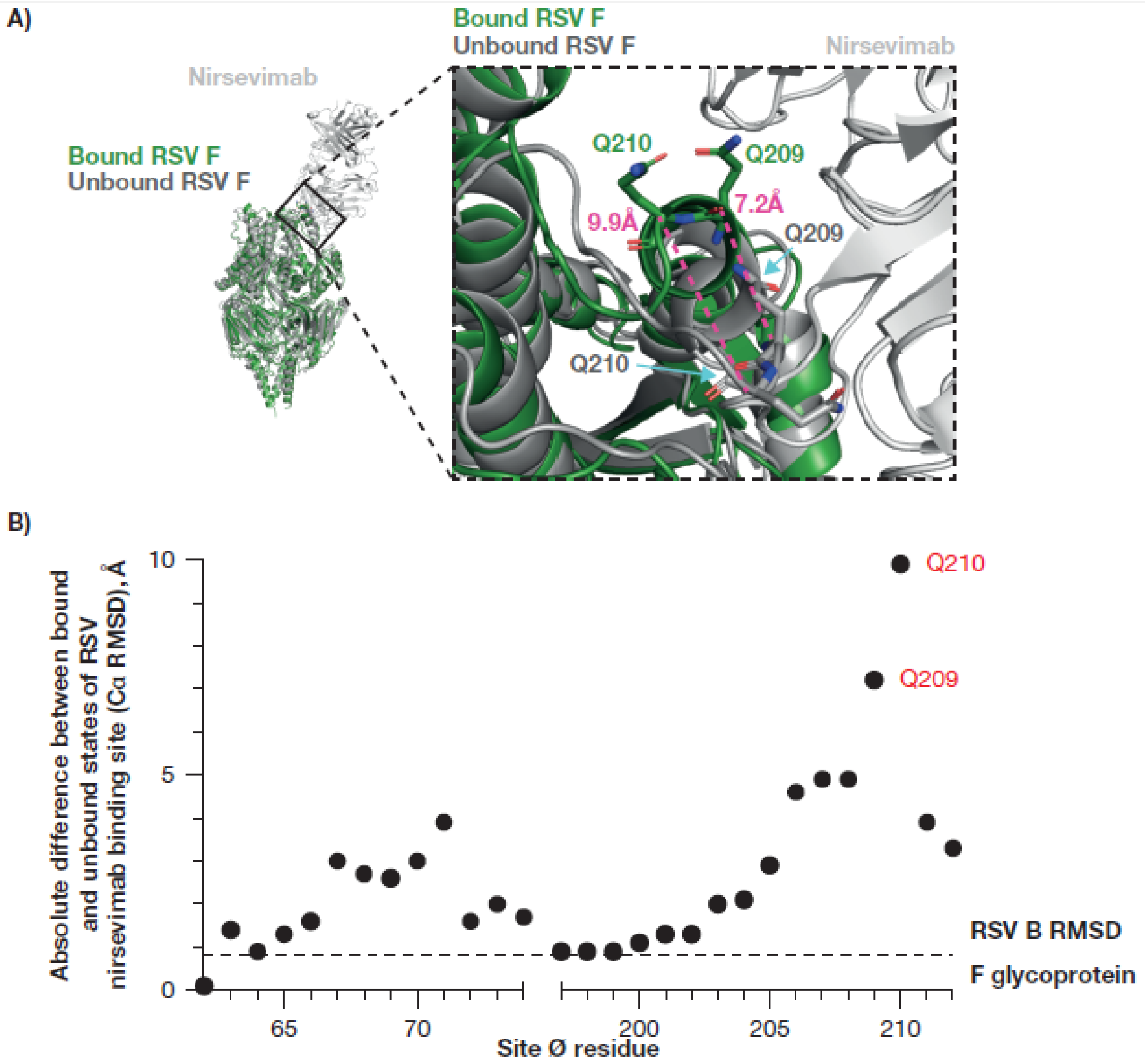

C)

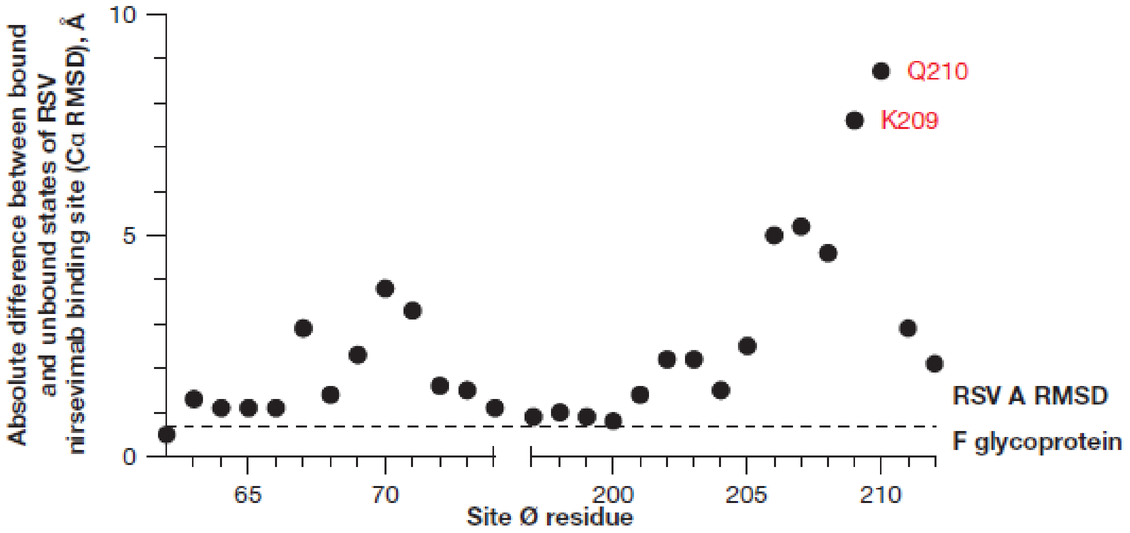

In (**A**) RSV B PDB ID: 5UDE[1]; nirsevimab PDB ID: 5UDD[1]. Site Ø residues Q209 and Q210 in both states are shown as sticks. Light blue arrows indicate the position of Q209 and Q210 in an unbound conformation. The measurements of Q209 and Q210 Cα atoms from unbound to bound states are shown as pink dashed lines, with the distance in angstroms also colored pink. Nirsevimab heavy and light chains are colored light gray. In (**B**) bound F glycoprotein RSV B monomers PDB ID: 5UDD[1]; unbound F glycoprotein RSV B monomers PDB ID: 5UDE[1]. In (**C**) bound F glycoprotein RSV A monomers PDB ID: 5UDC[1]; unbound F glycoprotein RSV B monomers PDB ID: 4MMU[2]. The RMSDs of each site Ø residue between bound and unbound states, as measured by Cα atom distance, were plotted following structural alignment. Residues with the largest conformational changes (Q209, K209, and Q210) are labeled with red text. Black dashed lines along the Y-axis indicate the all-atom RMSD of bound-unbound monomer alignment, with the value in (**A**) equal to 0.8 Å, and the value in (**B**) equal to 0.7 Å. All structural alignment, image capture, and distance measurements were conducted in PyMOL.

PDB, Protein Data Bank; RMSD, root mean squared distance; RSV, respiratory syncytial virus.

Supplementary Fig. S5 | Proposed mechanism of decreased RSV B neutralization potency with nirsevimab binding site substitutions K68E and N208S.

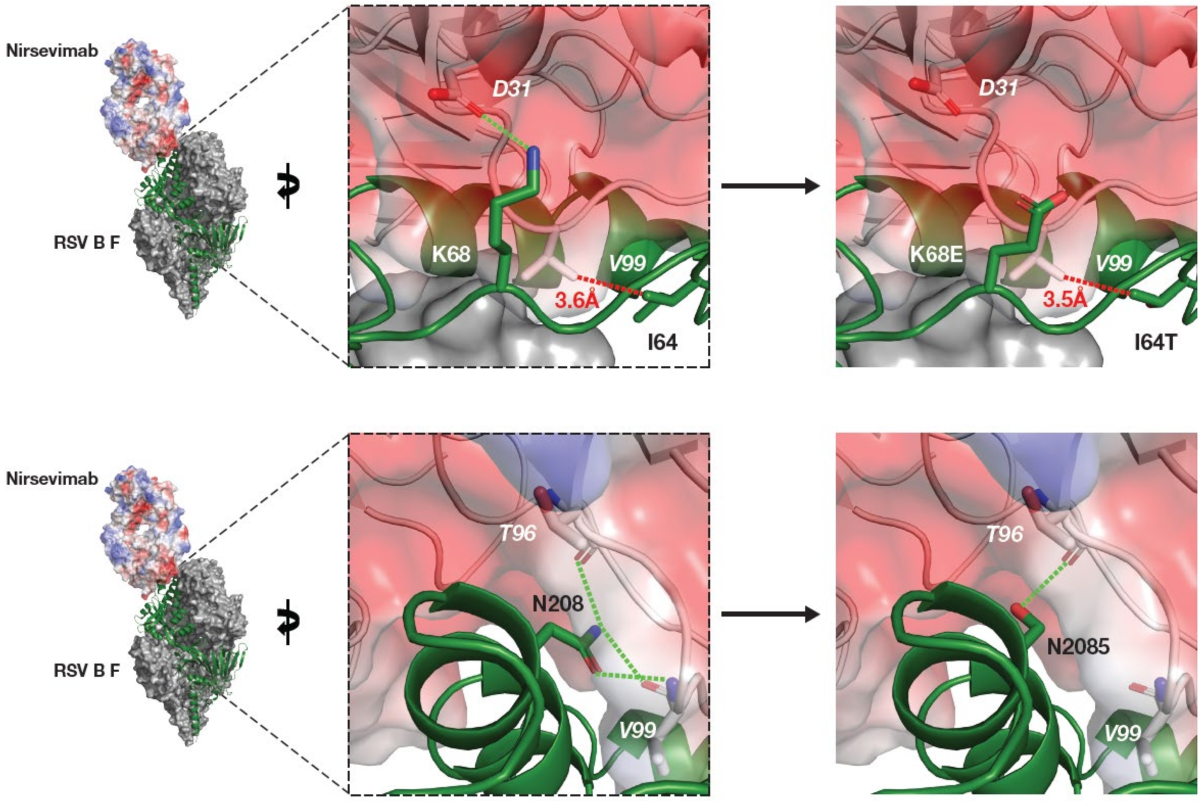

Red indicates electrostatics of the negatively charged pocket on nirsevimab. Green dashed lines indicate polar contacts. Red dashed lines are distance measurements.

F, fusion protein; RSV, respiratory syncytial virus

Independent Ethics Committees/Institutional Review Boards consulted

Site Number	Name/Address of IRB/IEC
**Phase 2b**	
2002923	Pharma Ethics 123 Amcor Road Lyttelton Manor Centurion Pretoria, Gauteng
2003359	MetroHealth Medical Center IRB 2500 MetroHealth Dr. Rammelkamp Bldg. Room 103, Cleveland, Ohio
2002934	CEP Investiga - Instituto de Pesquisas Avenida Romeu Tortima, 739 - Cidade Universitária Campinas, Sao Paulo
2002970, 2003091, 2003395, 2003007, 2003356, 2003405, 2003355, 2003354, 2003004, 2003353, 2002971, 2003124, 2003350, 2003394, 2003092, 2003348, 2003078, 2002974, 2003036, 2003346, 2003340, 2003441, 2003167, 2003338, 2003337, 2003442, 2003068, 2003038, 2003342, 2003335, 2003399, 2003086, 2003444, 2003400, 2003332, 2002976, 2003402, 2003347, 2003403, 2003125, 2003329, 2003336, 2003079, 2003401, 2003407	Copernicus Group IRB 5000 CentreGreen Way Suite 200, Cary, North Carolina
2002935	CEP da Universidade Federal de Minas Gerais Avenida Presidente Antonio Carlos 6627 Unidade Administrativa II Belo Horizonte Minas Gerais Andrade
2002947	CEIC de Galicia C/ San Lázaro, s/n Secretaria Xeral. Conselleria de SanidadeDirección Santiago de Compostela La Coruña Ares
2002948	CEIC de Galicia C/ San Lázaro, s/n Secretaria Xeral. Conselleria de SanidadeDirección Santiago de Compostela La Coruña Arimany Montaña,
2003358	Medical University of South Carolina IRB 19 Hagood Avenue 6th floor, Suite 601 Charleston South Carolina
2002918	Wits Health Consortium 31 Princess of Wales Terrace Parktown Johannesburg Gauteng
2002998	Comite Etico Cientifico del Servicio de Salud Metropolitano Sur Santa Rosa 3453, Piso 1 San Miguel Santiago
2003034	CESC della Provincia di Padova Presso Azienda Ospedaliera di Padova_Via Giustiniani 1 Padova
2002939	CEP da Faculdade de Ciências Médicas e da Saúde de Juiz de Fora SUPREMA/MG Alameda Salvaterra, 200 Bairro Salvaterra Juiz de Fora Minas Gerais Bastos
2003060	CEP da Faculdade de Medicina de Botucatu - UNESP/SP Distrito de Rubião Junior Botucatu Sao Paulo
2003000	Comitato Etico per la Sperimentazione Clinica delle Provincie di Verona e Rovigo P.le Stefani, 1 Verona
2002919	Pharma Ethics 123 Amcor Road Lyttelton Manor Centurion Pretoria

Site Number	Name/Address of IRB/IEC
2002910	Monash Health Human Research Ethics Committee (RGO) Level 2, I Block Clayton Victoria
2002953	Comité Etico Científico Servicio de Salud Valdivia Maipú 550, oficina 307 Valdivia
2002920	University of Stellenbosch Ethics Committee Faculty of Health Sciences Francie van Zijl Drive Tygerberg Cape Town Western Cape
2002956	Comité Ético Científico Servicio de Salud Metropolitano Central Victoria Subercaseaux 381, piso 4 Santiago
2003352	UTHSC IRB Office 910 Madison Suite 600 Memphis Tennessee
2003118	Memorial Health Services Research Council 2801 Atlantic Avenue Attn Research Administration Long Beach California
2002972	SUNY IRB 750 East Adams Street CWB 218G Syracuse New York
2003277	McGill University Health Center-Research Ethics Board 2155 Guy Street 2nd Floor, Room 231 Montreal Quebec
2002921	Pharma Ethics 123 Amcor Road Lyttelton Manor Centurion Pretoria Gauteng
2002973, 2003061, 2003069, 2003093, 2003331, 2003447	WIRB 1019 39th Avenue SE Suite 120 Puyallup Washington
2002905	Comité de Ética en Investigación Científica. Hospital Pediátrico Dr. Humberto Notti Bandera de Los Andes 2603 Villa Nueva Guaymallén Mendoza
2002967	R&D University Hospital Southampton NHS Foundation Trust Tremona Road, Level E, Laboratory & Pathology Block, SCBR - MP 138 Southampton, Hampshire
2003320	R&D - Brighton and Sussex University Hospitals Royal Sussex County Hospital Level 5 Thomas Kemp Tower Eastern Road, Brighton East Sussex
2003065	Azienda Ospedaliera Città della Salute e della Scienza di Torino Corso Bramante 88/90. Torino
2002940	Comitê de Ética em Pesquisa em Seres Humanos do Instituto de Medicina Integral Professor Fernando F Rua dos Coelhos, 300 - Boa Vista Recife Pernambuco Gomes
2002922	University of Cape Town HREC Faculty of Health Sciences Research EC E52-24 Old Main Building Groote Schuur Hospital, Observatory Cape Town Western Cape
2002941	CEP da Universidade Luterana do Brasil - ULBRA Farroupilha, 8001 - Prédio 14 - Sala 224 Bairro São José Canoas Rio Grande do Sul
2003319	R&D - Alder Hey Children's NHS Foundation Trust Eaton Road Liverpool Merseyside
2002968	R&D South West London and St George's Mental Health NHS Trust Department of Mental Health, St George's, University of London, 6th Floor, Hunter Wing, Cranmer Terrace London Greater London
2002924	Eticka komise IKEM a FTNsP Videnska 800 Praha 4 - Krc
2003343	Sharp Healthcare IRB 7930 Frost St Suite 300 San Diego California

Site Number	Name/Address of IRB/IEC
2003341	Winthrop-University Hospital IRB 222 Station Plaza North Suite 521 Mineola New York
2002943	Comitê de Ética em Pesquisa em Seres Humanos do Hospital Pequeno Príncipe Rua Desembargador Motta, 1070 6º andar, sala do NUPE Curitiba Paraná
2003339	Marshall University Office of Research Integrity One John Marshall Drive Huntington West Virginia
2002937	Wits Health Consortium 31 Princess of Wales Terrace Parktown Johannesburg Gauteng
2002950	CEIC de Galicia C/ San Lázaro, s/n Secretaria Xeral. Conselleria de SanidadeDirección Santiago de Compostela La Coruña Martinon
2002944	CEP da Universidade de Passo Fundo/RS Universidade de Passo Fundo - BR 285, Bairro São José Passo Fundo Rio Grande do Sul
2003011	Ann & Robert H. Lurie Children’s Hospital of Chicago Institutional Review Board 225 E. Chicago Avenue Box 59 Chicago Illinois
2003334, 2002975	Chesapeake IRB 7063 Columbia Gateway Drive Suite 110 Columbia Maryland
2003067	Cincinnati Children's Hospital Medical Center IRB 3333 Burnet Ave. MLC 5020 Cincinnati Ohio
2002954	Comité Ético-Científico Servicio de Salud Metropolitano Sur Oriente Av Concha y Toro 3459 Puente Alto Santiago
2003333	Childrens Hospital of Los Angeles-Committee on Clinical Investigations IRB 4650 Sunset Blvd Mail Stop #23 Dr. Andreas Reiff Los Angeles California
2003280	McGill University Health Center-Research Ethics Board 2155 Guy Street 2nd Floor, Room 231 Montreal Quebec
2003005	University of Texas at San Antonio IRB One UTSA Circle MS 4.01.82 San Antonio Texas
2002951	CEIC de Galicia C/ San Lázaro, s/n Secretaria Xeral. Conselleria de SanidadeDirección Santiago de Compostela La Coruña
2002911	Royal Children's Health Services Human Research Ethics Committee (RGO) 50 Flemington Road Parkville Victoria
2002938	Pharma Ethics 123 Amcor Road Lyttelton Manor Centurion Pretoria Gauteng
2003257	Comité Ético-Científico Servicio de Salud Viña del Mar-Quillota Calle Limache #1307 Esquina Peñablanca 2º Piso Viña del Mar Quilodran
2003035	Comitato Etico Regionale della Liguria Largo Rosanna Benzi 10 Farmacia Ospedaliera Genova
2002912	Princess Margaret Hospital for Children Ethics Committee Princess Margaret Hospital Entrance No 6, Hamilton Street Subiaco Western Australia
2003330	Arnold Palmer Medical Center Institutional Review Board 1401 Kuhl Avenue MP #21 Research Department Orlando Florida
2003328	University of Nebraska Medical Center IRB 987830 Nebaska Medical Center, Omaha Nebraska

Site Number	Name/Address of IRB/IEC
2002969	R&D - CRN Thames Valley and South Midlands 1st Floor, Manor House The John Radcliffe Hospital, Headley Way Headington Oxford Oxfordshire
2002926	Eticka komise Ustav pro peci o matku a dite Podolske nabrezi 157/36 Praha 4 - Podoli
2002909	Comité Hospitalario de Etica Necochea 675 Bahia Blanca Buenos Aires
2003274	Comité d'Ethique du CHU Ambroise Paré Boulevard Kennedy 2 Mons Van
2002955	Comité de Ética de Investigación en Seres Humanos Av. Independencia 1027, Independencia Santiago Vargas
2003009	Creighton University IRB 2500 California Plaza IRB-Biomedical Omaha Nebraska
2002966	R&D University Hospitals Bristol NHS Foundation Trust Education & Research Centre Level 3 Upper Maudlin Street Bristol Avon
2002999	Comite Etico Cientifico del Servicio de Salud Metropolitano Sur Santa Rosa 3453, Piso 1 San Miguel Santiago Villena
2002927	Eticka komise Nemocnice Havlickuv Brod Husova 2624 Havlickuv Brod Weberova,
2003448	Oklahoma University Health Sciences Center 1105 North Stonewall Avenue Oklahoma City Oklahoma
2003406	Connecticut Children's Medical Center IRB 282 Washington Street. Suite 2 K. Hartford Connecticut
2002946	University of Cape Town HREC Faculty of Health Sciences Research EC E52-24 Old Main Building Groote Schuur Hospital, Observatory Cape Town Western Cape
**MELODY**	
2004023, 2004025, 2004027, 2004028, 2004030, 2004031, 2004032, 2004118, 2004236, 2004237, 2004239, 2004240, 2004243, 2004253, 2004255, 2004256, 2004258, 2004259, 2004260, 2004261, 2004263, 2004264, 2004267, 2004268, 2004278, 2004279, 2004280, 2004291, 2004292, 2004293, 2004314, 2004315, 2004316, 2004319, 2004323, 2004340, 2004345, 2004376, 2004386, 2004389, 2004394, 2004409, 2004613, 2004614, 2004615, 2004618, 2004624, 2004634, 2004650, 2004652, 2004656, 2004657, 2004664, 2004677, 2004679, 2004680, 2004690, 2004697, 2004699, 2004700, 2004702, 2004746, 2004873, 2005604, 2005605, 2005606	WCG IRB, 212 Carnegie Center, Suite 301, Princeton, NJ 08540, USA

Site Number	Name/Address of IRB/IEC
2004026	The University of Oklahoma, Institutional Review Board for the Protection of Human Subjects, 1105N. Stone wall Avenue, Oklahoma City, OK73117(FWA 007961)
2004029	Nemours Office of Human Subjects Protection, Nemours/Alfred I. duPont Hospital for Children, 1600 Rockland Road, Wilmington, DE 19803
2004036	University of Cape Town Human Research Ethics Committee, DEPARTMENT OF PAEDIATRICS AND CHILD HEALTH, RED CROSS WAR MEMORIAL CHILDREN'S HOSPITAL, KLIPFONTEIN ROAD, RONDEBOSCH, 7700
2004039	Stellenbosch University Human Research Ethics Committee, Stellenbosch University, Private Bag X1, Matieland, 7602, Stellenbosch, South Africa
2004043	Servicio De Salud Metropolitano Sur Oriente Comite Etico-Cientifico, Av. Concha y Toro 3459 – Paradero 30, Vic. Mackenna
2004098	UNIVERSIDAD DE CHILE [University of Chile] – FACULTAD DE MEDICINA, HUMAN RESEARCH ETHICS COMMITTEE, Av. Libertador Bernardo O'Higgins 1058, Santiago de Chile
2004103	Comitato Etico per la Sperimentazione Clinica delle Provincie di Verona e Rovigo,P.le Stefani, 1, Verona,37126
2004111	1 Military Hospital Human Research Ethics Committee, Department of Neurology Private bag X 1026 Thaba Tswane 0143
2004117	Dept of health of Chernivtsi city council, Communal Medical Institution City Clinical Childrens' Hospital, 4 Bukovynska St, Chernivtsi, 58001
2004132	Independent Ethics Committee for Clinical Pharmacology Trials, Drug and Pharmacology Studies Foundation, LA FUNDACIÓN DE ESTUDIOS FARMACOLOGICOS Y DE MEDICAMENTOS, Pte. J. E. Uriburu 774 1º Piso Ciudad Autónoma de Buenos Aires (C1027AAP), Argentina
2004178	Landesärztekammer Baden-Württemberg, Ethik-Kommission, Liebknechtstr. 33, 70565 Stuttgart
2004182	Ethik-Kommission der Bayerischen Landesärztekammer, Mühlbaurstr.16, D-81677 München
2004185	Ethik-Kommission an der Medizinischen Fakultaet der Universitaet Leipzig,Kaethe-Kollwitz-Strasse 82,Haus: Karl-Sudhoff-Institut Leipzig,04109
2004222,	Ege University Ethics Committee,Ege Universitesi Tip Fakultesi,Klinik Arastirmalar Etik Kurulu Izmir,35100
2004227	Ministy of Health of Ukrain, Communal Non-Commercial enterprise Saint Zinaida Children's Clinical Hospital of Sumy City Council, 28 Troiiska st, Sumy, 40022
2004229	Vinnytsia regional Children's Clinical Hospital, 108 Khmelnytske shose st, Vinnytsia, 21000. Medical Ethics Commission
2004233	Universidad Pontificia Bolivariana, Calle 78 B No. 72 A 109
2004238	Institutional Review Board, Ann & Robert H. Lurie Children's Hospital of Chicago, 25 East Chicago Avenue, Chicago, Illinois
2004241	Cincinnati Children's Hospital Institutional Review Board, 3333 Burnet Avenue | MLC 7040 | Cincinnati, OH 45229

Site Number	Name/Address of IRB/IEC
2004281	MetroHealth Institutional Review board, 2500 MetroHealth Drive, Cleveland Ohio 44109
2004294	State Institution Academician O.M Lukyanova Institute of Pediatrics, obstetrics and gynecology of national academy of medical sciences of Ukraine, 8 P.Mayborody str Kyiv, 04050
2004295	CORPORACIÓN CIENTÍFICA PEDIÁTRICA, BIOMEDICAL RESEARCH ETHICS COMMITTEE, Calle 5 B5 No. 37 bis - 28
2004296	Ministerio de Salud, Servicio de Salud Valdivia, Scientific Ethics Committee, V. Pérez Rosales 560 - Edificio Prales - Oficina 307 - Piso 3
2004300	Servicio de Salud Metropolitano Norte, Research Ethics Committee, 272, Calle Maruri 8380000 Independencia Metropolitana de Santiago
2004304	UNIVERSIDAD CES, Calle 10A No. 22 - 04 El Poblado
2004310	Creighton University office of the provost Research Compliance, 2500 California Plaza Omaha, NE 68178-0001
2004322	Communal Non-Commercial enterprise of Kharkiv Regional Council Regional Children's clinical hospital, 5 Ozeryanska st Kharkiv, 61093
2004338	Odesa Regional State administration, department of health, communal enterprise, Odesa regional Children's clinical hospital, 3 Ac Vorobiov st, Odes-31, 65031
2004341	Medical University of South Carolina, 179 Ashley Ave, Charleston, SC 29425
2004351	MUHC Centre for Applied Ethics, 5100, boul. de Maisonneuve Ouest, 5th floor, Office 576, Montréal, Québec, H4A 3T2
2004359	Ethikkommission der Landesärztekammer Rheinland-Pfalz Deutschhausplatz 3 55116 Mainz
2004365	MUHC Centre for Applied Ethics, 5100, boul. de Maisonneuve Ouest, 5th floor, Office 576, Montréal, Québec, H4A 3T2
2004372	COMITÉ DE ÉTICA EN INVESTIGACIÓN VIT, Calle 24 N° 3-02 este
2004391	University of Nebraska Medical Center, 42nd and Emile Streets, Omaha, NE 68198, 402-559-4000
2004396	COMITATO ETICO DELLA FONDAZIONE POLICLINICO UNIVERSITARIO AGOSTINO GEMELLI IRCCS UNIVERSITÀ CATTOLICA DEL SACRO CUORE
2004400	Research Ethics Committee of the Health Sciences Department of the Universidad del Norte, Apartados Aéreos 1569 - 51820, Km. 5 vía Puerto Colombia
2004404	Federico Gomez Children's hospital of Mexico, National Institute of Health research office
2004616	Stony Brook University, Health Sciences Center Room 031, Stony Brook, NY 11794-8111
2004623	UBC C&W Research Ethics Board A2-141A, 950 West 28th Avenue Vancouver, BC V5Z 4H4
2004626	Soroka University Medical Center, Itzhak Rager Blv. Beer Sheva 8458900
2004632	Japanese Red cross Maebashi Hospital IRB 138-Asakuramachi, Maebashi-Shi Gunma

Site Number	Name/Address of IRB/IEC
2004633	Ethics Commission at Communal Institution Dnipro City Children's Clinical Hospital No 5 of Dnipro City Council, 5 ivana Akinfiieva st, Dnipro 49027 Ukraine
2004648	Independent Ethics Committee for Clinical Pharmacology Trials, Drug and Pharmacology Studies Foundation, LA FUNDACIÓN DE ESTUDIOS FARMACOLOGICOS Y DE MEDICAMENTOS, Pte. J. E. Uriburu 774 1º Piso Ciudad Autónoma de Buenos Aires (C1027AAP), Argentina
2004658	Nationwide Children's IRB, Nationwide Children’s Hospital, 700 Childrens Drive, Columbus, OH 43205
2004660	Yokosuka Kyosai Hospital IRB, 1-16 Yonegahamadori, Yokosuka Kanagawa
2004662	The University of Tennessee, Health Science Centre Institutional Review Board, 910 Madison Avenue, Suite 600, Memphis, TN 38163
2004667	Conjoint Health Research Ethics Board, Research Services Office, 2500 University Drive, NW, Calgary AB T2N 1N4
2004668	Jimbo Orthopedic Surgery, Institutional Review Board, 5-38-41, Honcho Koganei-shi, Tokyo
2004669	State Social Enterprise, HOSPITAL MENTAL DE ANTIOQUIA, [Antioquia Psychiatric Hospital], Calle 38 55-310 Bello-Colombia
2004670	NHO Okayama Medical Center IRB, Kita-ku Tamasu 1711-1, Okayama-shi, Okayama-Ken, Japan
2004671	Kawasaki Municipal Hospital Institutional Review Board, 12-1, Shinkawa-dori, Kawasaki-ku, Kawasaki-shi, Kanagawa
2004672	Laniado Hospital, 16, deuteronomy haim st., kiryat sanz, netanya, 42150
2004678	Marshfield Clinic Research Institiute Institutional Review Board, 1000N, Oak Ave, Marshfield, WI 54449-5790
2004681	Human Research Ethics Committee, Fundación Hospital Infantil Universitario de San José, Carrera 52 No. 67 A-71 PBX: 4377540
2004687	Fukuyama City Hospital Institutional Review Board, 5-23-1 Zao-cho, Fukuyama-shi, Hiroshima
2004688	KKR Sapporo Medical Center IRB, 6-3-40 Hiragishi 1-jo Toyohira-ku, Sapporo-shi, Hokkaido
2004708	EMORY UNIVERSITY Institutional Review Board, 201 Dowman Dr, Atlanta, GA 30322, United States
2004747, 2004749	Navajo Nation Human Research Review Board, Navajo Division of Health, P. O. Box 1390, Window Rock, AZ 86515
2004748	Johns Hopkins Bloomberg School Of Public Health, Institutional Review Board Office, 615 N. Wolfe Street / Room E1100 Baltimore, Maryland 21205-2179
2004768	Samsung Medical Center Institutional Review Board, (06351) 81 Irwon-Ro Gangnam-gu. Seoul, Korea
2004769	Yonsei University Health system, Severance Hospital, Institutional review Board, Yonsei-ro 50-1, Seodaemun-gu, Seoul, 03722
2004797	human research Protection Program of Korea University medical Center 123 Jeokgeum-ro (Gojan-dong) Danwon-gu, Ansan-si, Gyeonggi-do, 15355

Site Number	Name/Address of IRB/IEC
2004798	Inha University Hospital Institutional Review Board, 27 Inhang-ro, Jung-gu, Incheon
2004800	Yonsei University Gangnam Severance Hospital, IRB, 2nd Floor, 235 Dogok-ro, Gangnam-gu, Seoul 06230
2005029	Fukui-ken Saiseikai Hospital Institutional Review Board, 7-1 Funabashi, Wadanaka-cho, Fukui-shi, Fukui-Ken
2005030	Institutional Review Board of Okayama City General Medical Center Okayama City Hospital, 3-20-1 Kitanagaseomotemachi, Kita-ku, Okayama-shi, Okayama
2005031	Local Independent Administrative Corporation, Hiroshima City Hospital Organization, Hiroshima City Hiroshima Citizens Hospital Institutional Review Board, 7-33 Motomachi, Naka-ku, Hiroshima-shi, Hiroshima
2005032, 2005034	Review Board of Human Rights and Ethics for Clinical Studies Institutional Review Board 13-2 Ichibancho, Chiyoda-ku, Tokyo
2005033	Aijinkai Takatsuki General Hospital IRB, 1-3-13 Kosobe-cho, Takatsuki, Osaka
2005035	Japanese Red Cross Shizuoka Hospital Institutional Review Board, 8-2 Otemachi, Aoi-ku, Shizuoka-shi, Shizuoka
2005036	JA Shizuoka Kosei Hospital Institutional Review Board, 23 Kitabancho, Aoi-ku, Shizuoka-shi, Shizuoka
2005037	Hiroshima Red Cross Hospital & Atomicbomb Survivors Hospital Institutional Review Board, 1-9-6 Sendamachi, Naka-ku, Hiroshima-shI
2005038	NHO Shikoku Medical Center for Children and Adults Institutional Review Board, 2-1-1, Senyucho, Zentsuji-shi, Kagawa, Japan
2005039	Daido Hospital Institutional Review Board, 9 Hakusuicho, Minami-ku, Nagoya, Aichi
2005049	Nagoya Ekisaikai Hospital IRB, 4-66 Shonen-Cho, Nakagawa-ku, Nagoya-Shi, Aichi
2004272, 2004044	Multicentricka eticka komise IKEM a TN,Videnska 800, Praha,140 59
2004402, 2004116	Etikprövningsmyndigheten,Box 2110,SE-750 02 Uppsala,SE-750 02
2004249, 2004298	Ethikkommission der Medizinischen Universität Graz,Auenbruggerplatz 2, Graz,8036
2004373, 2004327	Child and Adolescent Health Service (HREC), Office 5E, Perth Children's Hospital,15 Hospital Avenue Nedlands,6009
2004401, 2004399,	Ethics Committee for Multicenter Trials,8 Damyan Gruev Str., Sofia,1303
2004887, 2004896	Hospital District of Southwest Finland, Joint Municipal Authority, Ethics Committee, Turku University Hospital, T-Hospital, 6th Floor, Board meeting room A 607
2004217, 2004109, 2004216	Wits Health Consortium,31 Princess of Wales Terrace, Parktown Johannesburg, 2193
2004212, 2004106, 2004214, 2004336	Ethical Council at the MoH of RF,3 Rakhmanovsky Pereulok, Moscow,127994

Site Number	Name/Address of IRB/IEC
2004335, 2004405, 2004048, 2004105	Northern B Health and Disability Ethics Committee,20 Aitken Street, Ministry of Health, Ethics Department, Reception - Ground Floor, Thorndon, Wellington,6011
2004034, 2004108, 2004110, 2004712	Pharma Ethics Independant Research Ethics committee,123 Amcor Road, Lyttelton Manor Pretoria,0157
2004395, 2004204, 2004277, 2004710	Lithuanian Bioethics Committee, Algirdo g. 31, Vilnius,LT-03219
2004355, 2004384, 2004682, 2004689, 2004383	NRES Committee South Central - Berkshire, South West REC Centre, Level 3, Block B Bristol,BS1 2NT
2004273, 2004045, 2004046, 2004099, 2004047, 2004274	Research Ethics Committee of the National Institute for Health Development, Hiiu 42, Tallinn,11619
2004380, 2004199, 2004331, 2004202, 2004198, 2004302	Ethics Committee for Clinical Trials of Medicinal Products, Aizkraukles street 21 - 113, Riga,LV1006
2004867, 2004868, 2004869, 2004870, 2004871, 2004872	Dr Jose Renan Esquivel Children's hospital, Panama Ave, Balboa, Calle 34 Research Bioethics Committee
2004033, 2004112, 2004113, 2004114, 2004115, 2004218, 2004219, 2004311, 2004333, 2004344, 2004363, 2004369, 2004382, 2004385, 2004406, 2004675, 2004407, 2005603	Hospital Universitario Clinico San Carlos,Puerta G - Planta 4ª Norte,C/ Profesor Martin Lagos, s/n Madrid, 28040
2004674, 2004371, 2004049, 2004334, 2004206, 2004381, 2004205, 2004208, 2004350, 2004305	Komisja Bioetyczna przy Okregowej Izbie Lekarskiej w Rzeszowie,ul. Jana Dekerta 2, Rzeszów, 35-030
2004629, 2004231, 2004270, 2004299, 2004320, 2004398, 2004320	O.L.V. Ziekenhuis, Moorselbaan 164, Aalst,9300
2004303, 2004234, 2004325, 2004339, 2004343, 2004639, 2004324	Ethics Committee for Clinical Trials,8, Damyan Gruev Str, Sofia, 1303
2004654, 2004100, 2004232, 2004374, 2004378, 2004646, 2004653, 2004676, 2005602	Comité de Protection des Personnes Ile de France VIII,Hôpital Ambroise Paré, 9 avenue Charles de Gaulle Boulogne Billancourt, 92100
2004742, 2004741, 2004313, 2004743, 2004611, 2004312, 2004644	Varsinais-Suomen sairaanhoitopiiri Eettinen toimikunta, Kiinamyllynkatu 4-8,PL 52 Turku, 20520

References

1. Zhu Q. et al. A highly potent extended half-life antibody as a potential RSV vaccine surrogate for all infants. *Sci Transl Med* **9**, eaaj1928 (2017).

2. McLellan J. S. et al. Structure-based design of a fusion glycoprotein vaccine for respiratory syncytial virus. *Science* **342**, 592-598 (2013).
