# Supplementary material for: Molecular and phenotypic characteristics of RSV infections in infants during two nirsevimab randomized clinical trials
Source: Nat Commun. 2023 Jul 19;14:4347. doi: 10.1038/s41467-023-40057-8 (PMC10356750; doi:10.1038/s41467-023-40057-8)
Supplement: Supplementary file 3 — Reporting Summary [file 41467_2023_40057_MOESM3_ESM.pdf]

nature portfolio

Corresponding author(s): Elizabeth J Kelly

Last updated by author(s): Jul 3, 2023

Reporting Summary

Nature Portfolio wishes to improve the reproducibility of the work that we publish. This form provides structure for consistency and transparency in reporting. For further information on Nature Portfolio policies, see our Editorial Policies and the Editorial Policy Checklist.

Statistics

For all statistical analyses, confirm that the following items are present in the figure legend, table legend, main text, or Methods section.

n/a	Confirmed	
☐	☒	The exact sample size (*n*) for each experimental group/condition, given as a discrete number and unit of measurement
☐	☒	A statement on whether measurements were taken from distinct samples or whether the same sample was measured repeatedly
☐	☒	The statistical test(s) used AND whether they are one- or two-sided<br>*Only common tests should be described solely by name; describe more complex techniques in the Methods section.*
☒	☐	A description of all covariates tested
☐	☒	A description of any assumptions or corrections, such as tests of normality and adjustment for multiple comparisons
☐	☒	A full description of the statistical parameters including central tendency (e.g. means) or other basic estimates (e.g. regression coefficient) AND variation (e.g. standard deviation) or associated estimates of uncertainty (e.g. confidence intervals)
☐	☒	For null hypothesis testing, the test statistic (e.g. *F*, *t*, *r*) with confidence intervals, effect sizes, degrees of freedom and *P* value noted<br>*Give P values as exact values whenever suitable.*
☒	☐	For Bayesian analysis, information on the choice of priors and Markov chain Monte Carlo settings
☒	☐	For hierarchical and complex designs, identification of the appropriate level for tests and full reporting of outcomes
☒	☐	Estimates of effect sizes (e.g. Cohen's *d*, Pearson's *r*), indicating how they were calculated

Our web collection on statistics for biologists contains articles on many of the points above.

Software and code

Policy information about availability of computer code

Data collection	No custom software or algorithms were used for data collection
Data analysis	Molecular Operating Environment (MOE) 2020.09 was used for in silico mutagenesis (Figure 7)<br>PyMOL Version 2.2.2 was used to visualize RSV/nirsevimab binding (Supplementary Figure 3)<br>Seq2Logo-2.0 (https://services.healthtech.dtu.dk/service.php?Seq2Logo-2.0) was used to generate Weblogos/Sequence Logos (Figure 3)<br>R version 4.0.4 (https://www.r-project.org/) was used for graphical analysis and statistical summary of pharmacokinetic data<br>Graphpad Prism version 9.4.1 was used for fitting a four-parameter logistics model for the serum neutralization assays<br>Graphpad Prism version 9.4.0 was used for calculating root mean squared distance measurements

For manuscripts utilizing custom algorithms or software that are central to the research but not yet described in published literature, software must be made available to editors and reviewers. We strongly encourage code deposition in a community repository (e.g. GitHub). See the Nature Portfolio guidelines for submitting code & software for further information.

Data

Policy information about availability of data

All manuscripts must include a data availability statement. This statement should provide the following information, where applicable:

- Accession codes, unique identifiers, or web links for publicly available datasets
- A description of any restrictions on data availability
- For clinical datasets or third party data, please ensure that the statement adheres to our policy

Crystal structure data are available from the Research Collaboratory for Structural Bioinformatics Protein Data Bank as follows:
RSV F B9320 unbound (PDB ID: 5UDE): https://www.rcsb.org/structure/5UDE
RSV F B9320 bound to nirsevimab (PDB ID: 5UDD): https://www.rcsb.org/structure/5UDD
RSV F A2 unbound (PDB ID: 4MMU): https://www.rcsb.org/structure/4MMU
RSV F A2 bound to nirsevimab (PDB ID: 5UDC): https://www.rcsb.org/structure/5UDC
Clinical isolates selected as references at the start of nirsevimab clinical development were available from GenBank® (RSV A-NLD-13-005275: accession code KX858757.1 [https://www.ncbi.nlm.nih.gov/nuccore/KX858757]; RSV B-NLD-13-001273: accession code KX858756.1 [https://www.ncbi.nlm.nih.gov/nuccore/KX858756]). The raw DNA sequence data generated in this study have been deposited in the GenBank® database under accession code PRJNA989584 (https://www.ncbi.nlm.nih.gov/bioproject/PRJNA989584/).

Trial data are subject to controlled access to ensure commitment to the Responsible Data Sharing Principles as established by EFPIA (European Federation of Pharmaceutical Industries and Associations) and PhRMA (Pharmaceutical Research and Manufacturers of America) and guided by the Declaration of Helsinki. Any restrictions are related to ensuring the fulfillment of legal and ethical obligation to protect patients when using patient data to advance medical research. Data underlying the findings described in this manuscript will be made available within timelines required by country laws and may be obtained in accordance with AstraZeneca's data sharing policy described at https://astrazenecagrouptrials.pharmacm.com/ST/Submission/Disclosure.
Data for studies directly listed on Vivli can be requested through Vivli at www.vivli.org. Data for studies not listed on Vivli could be requested through Vivli at https://vivli.org/members/enquiries-about-studies-not-listed-on-the-vivli-platform/. AstraZeneca Vivli member page is also available outlining further details: https://vivli.org/ourmember/astrazeneca/. Data underlying the findings described in this manuscript may be obtained in accordance with AstraZeneca's data sharing policy described at https://astrazenecagrouptrials.pharmacm.com/ST/Submission/Disclosure.
Data for studies directly listed on Vivli can be requested through Vivli at www.vivli.org. Data for studies not listed on Vivli could be requested through Vivli at https://vivli.org/members/enquiries-about-studies-not-listed-on-the-vivli-platform/. AstraZeneca Vivli member page is also available outlining further details: https://vivli.org/ourmember/astrazeneca/.

Human research participants

Policy information about studies involving human research participants and Sex and Gender in Research.

Reporting on sex and gender

Both the Phase 2b and MELODY studies enrolled patients of either sex and no differences in outcomes were found.

Population characteristics

Baseline demographics and patient characteristics are reported in the results text. Mean age at randomization was 3.4 months in the full cohort of the phase 2b study and 3.0 months in full primary cohort MELODY for the population in this analysis.

Recruitment

Healthy infants ≤1 year of age upon entering their first full RSV season, with a gestational age of ≥29 weeks 0 days to <34 weeks 6 days (Phase 2b) or ≥35 weeks 0 days (MELODY), were enrolled across a full range of settings. Infants were excluded from the study if they were eligible to receive palivizumab (according to national or local guidelines), had a fever or acute illness within 7 days of randomization, or had history of respiratory syncytial virus disease or lower respiratory tract infection prior to or at the time of randomization. For inclusion in this specific analysis, participants were also required to have next generation sequencing- (NGS-) evaluable RSV isolates.

Together, the phase 2b and MELODY trials enrolled infants across 4 years in both the Northern and Southern Hemispheres in both inpatient and outpatient settings. The phase 2b study was performed at 164 sites in 23 countries; MELODY was performed in 160 sites in 21 countries:

Phase 2b study sites
Site # Centre address
2002892 SE Gyermekgyógy. Klinika Tuzolto utca 7-9, Budapest, 1094
2002893 Borsod-Abauj-Zemplen Megyei Ko Szentpeteri Kapu 72-76, Borsod-Abauj- Zemplen Megyei Korhaz es Egyetemi OktatoKorhaz, Miskolc, 3526
2002894 Bács-Kiskun Megyei Kórház Csecsemő- es Gyermekosztály, Nyíri u. 38, Kecskemét, 6000
2002897 Middlemore Hospital, 100 Hospital Road, Papatoetoe, Otahuhu, New Zealand 2025
2002898 Wellington Hospital, Level 8, CSB, Wellington hospital, Riddiford Street, Wellington, 6021
2002899 University Clinical Center, ul Clinical 1A, Gdansk, 80402
2002900 Univ Hosp No 2 in Bydgoszcz, ul Ujejskiego 75, Bydgoszcz, Kujawsko-Pomorskie 85168
2002901 University Children's Hospital, Department of Pediatrics, 265 Wielicka Street, Krakow, 30-663
2002903 Centrum Medyczne Plejady, Ul. Miłkowskiego 11A / 128, Krakow, 30-349
2002905 Hospital Pediatrico Dr. Humber, Bandera de los Andes 2603, Guaymallen Mendoza, Mendoza 5519
2002909 Hospital Italiano Regional del, Necochea 675, Bahía Blanca, Buenos Aires B8001HXM
2002911 Murdoch Childrens Research Ins, 50 Flemington Rd, Parkville, VIC 3052

2002912 Telethon Kids Institute, 100 Roberts Rd, Subiaco, WA 6008
2002918 Wits Clinical Research, 18 Eton Rd, Parktown, Johannesburg, Gauteng 2193
2002919 Dr F Bocking, Suite 110 Consultation Block, 331 Burger Street, Pietermaritzburg, KwaZulu Natal 3201
2002920 FAMCRU, Ward J8, Tygerberg Academic Hospital, Francie van Zijl Drive, Parow, Cape Town, Western Cape 7500
2002921 Egner, JG, 107 JB Marks Rd, Suite 111, Durban, KwaZulu-Natal 4091
2002922 Allergy Diagnostics & Clinical, University of Cape Town Lung Institute, George Street, Cape Town, Mowbray 7700
2002923 VX Pharma, 6 Mark Shuttleworth St, Unit U3-U7 The Enterprise Building, Pretoria, Gauteng 0087
2002924 Thomayerova, Nemocnice, Videnska 800, Praha 4, 14059
2002926 Ustav pro peci o matku a dite, Podolske nabrezi 157, Praha 4, 14710
2002927 Nemocnice Havlickuv Brod, Husova 2624, Havlickuv Brod, 580 22
2002928 SZTE Gyerm. Klinikai Kozpon, Koranyi Fasor 14-15, Szeged, 6720
2002929 Jahn Ferenc Del-Pesti Korhaz e, Koves u. 1, Budapest, 1204
2002930 Heim Pal Gyermekkorhaz, Madarasz Viktor u. 22-24, Budapest, 1131
2002931 Soproni Erzsébet Oktató Kórház, Győri út 15, Sopron, 9400
2002932 Kanizsai Dorottya Korhaz, Szekeres Jozsef u 2-8, Nagykanizsa, 8800
2002933 Magyar Honvédség EU Központ, Podmaniczky u. 111, Budapest, 1062
2002934 Allergisa Pesquisa Dermato-Cos, Avenida Dr Romeu Tórtima 452, Barão Geraldo, Campinas, Sao Paulo 13084- 791
2002937 Respiratory and Meningeal Path, Chris Hani Road, 11th Floor West Wing New Nurses Residence, Johannesburg, Gauteng 2013
2002938 Cape Allergy Asthma Clinic, Syringa House, 31 Wilderness Road, Claremont, Western Cape 7708
2002939 HMTJ - Hospital e Maternidade, Rua Doutor Dirceu de Andrade, 33, São Mateus, Juiz de Fora, Minas Gerais 36025-330
2002941 Hospital ULBRA Universitario, Avenida Farroupilha 8001, Prédio 21 - 2º andar, São José, Canoas, Rio Grande do Sul 92425-900
2002943 Hospital Infantil Pequeno Prín, Rua Desembargador Motta, 1070, Água Verde, Curitiba, Paraná 80250-060
2002944 Hospital São Vicente de Paulo, Rua Teixeira Soares 808, Centro, Passo Fundo, Rio Grande do Sul 99010-080
2002946 Red Cross Children's Hospital, Klipfontein Road, Rondebosch, Cape Town, 7700
2002947 Hospital Universitario La Paz, Paseo de la Castellana 261, Madrid, Madrid 28046
2002948 Hospital General de Catalunya, Pere i Pons 1, Servicio de Reumatologia, Sant Cugat del Valles, Barcelona 8190
2002950 Complejo Hospitalario Univers, Travesia da Choupana s/n, Pediatria, Santiago de Compostela, La Coruña 15706
2002951 Hospital Materno Infantil Carl, Avda Arroyo de los Angeles s/n, Pediatria, Málaga, Málaga 29011
2002953 Hospital Base Valdivia, Bueras 1003. Policlinico Respiratorio, oficina 5, Hospital Regional de Valdivia, Valdivia, 5090000
2002954 Unidad de Neonatologia Hosp, Esperanza 2150, San Ramon, Santiago, 8880465
2002955 Facultad de Medicina, Av Independencia 1027, Independencia, Santiago, 8380453
2002956 Hospital El Carmen Dr. Luis Va, Camino a Rinconada 1201, Pediatric Service, Maipu, 9250000
2002958 CHU Lyon - Hôpital Femme-Mère-59 Boulevard Pinel, Service Pharmacie, Bron, Rhone 69677
2002959 CHRU de Brest - Hôpital Morvan, 2 Avenue Foch, Brest, Finistere 29609
2002960 Hôpital du Bocage, 10 boulevard Maréchal de Lattre de Tassigny, Dijon, 21079
2002961 Groupe Hospitalier Pellegrin, Place Amelie Raba-Leon, Bordeaux, AQUITAINE 33000
2002962 SHATPPD Dr. Dimitar Gramatikov, 1 Alley Lilia str., Ruse, Ruse 7002
2002963 MHAT-Plovdiv AD, 234 Bulgaria Blvd, Plovdiv, 4002
2002964 Acibadem City Clinic Tokuda, 51B Nikola Vaptzarov Blvd, Sofia, 1407
2002966 Bristol Royal Hosp for Children, Paul O'Gorman Building, Upper Maudlin Street, Bristol, Avon BS2 8BJ
2002967 Southampton General Hospital, Tremona Road, Southampton, England SO16 6YD
2002968 St Georges University of London, Cranmer Terrace, Jenner Wing Level 0, Room 0.160, London, Greater London SW17 0RE
2002969 Oxford University HospitalNHF, Oxford Vaccine Group, Churchill Hospital, Old Road, Headington, Oxford, OX3 7EJ
2002970 Children's Healthcare of Atlanta, 1405 Clifton Rd NE, Main Pharmacy, Atlanta, GA 30322
2002971 Premier Health Research Center, 9317 Firestone Blvd, Downey, CA 90241
2002972 SUNY Upstate Medical University, 750 East Adams Street, 5400 University Hospital, Syracuse, NY 13210-2306
2002973 Children's Hospital and Region, Division of Infectious Disease, 4800 Sand Point Way NE #R-5441, Seattle, WA 98105
2002974 Ignatius Godoy, MD, 16660 Paramount Boulevard, Suite 206, Paramount, CA 90723
2002975 West Virginia University, 8 Medical Center Drive, Suite 2020, Morgantown, WV 26506
2002976 Emmaus Research Center, Inc., 408 S. Beach Blvd., Suite 111, Anaheim, CA 92804
2002994 UMHAT Sveti Georgi EAD, 15A Vasil Aprilov Blvd, Plovdiv, 4002
2002995 UMHAT Dr Georgi Stranski, 8A Georgi Kochev Blvd, Pleven, 5800
2002996 UMHAT Dr Georgi Stranski, 91 Gen Vladimir Vazov Str, Second Clinical Base, Pleven, 5800
2002997 UMHAT Sveti Georgi EAD, 66 Peshtersko Shose Str, Second Clinical Base, Plovdiv, 4002
2002998 Hospital El Pino, Av. Padre Hurtado 13560, San Bernardo, Santiago, 8053095
2003000 AO Universitaria Integrata, Piazzale A Stefani 1, Verona, VR 37126
2003002 Niepubliczny ZakBad Opieki Zdr, ul Wacława Jawoszka 3, 21-010 Łęczna, NZOZ Salmed, Leczna, 21- 010
2003003 Children's Hosp of Michigan, 3901 Beaubien St, Detroit, MI 48201
2003004 Advocate Hope Children's Hosp, Division Head, Pediatric Infectious Diseases, Advocate Children's Hospital, Dept of Pediatrics- Room 1404, 4440 West 95th Street, Oak Lawn, IL 60068
2003005 Univ. of Texas Medical Branch, 301 University Blvd, Research Building 6, 3rd Floor, Room 3.320C, Galveston, TX 77555
2003006 Nationwide Children's Hospital, CLINICAL RESEARCH SERVICES CENTER FOR CLINICAL AND TRANSLATIONAL RESEARCH, The Research Institute at Nationwide Children’s Hospital, 700 Children’s Drive, Tower, 6th floor, Columbus, OH

43205
2003007 Sarkis Clinical Trials, 611 NW 60th St, Suite C, Gainesville, FL 32607
2003008 The Childrens Hospital, 13123 E 16th Ave, Aurora, CO 80045
2003009 Creighton Univ Medical Center, Clinical Research Office, 7710 Mercy Road, Suite 228, Omaha, NE 6812441
2003010 Advocate Lutheran General, 1775 Dempster Street, Park Ridge, IL 60068
2003011 Ann & Robert H Lurie Children, 225 E Chicago Ave, Chicago, IL 60611
2003034 Azienda Ospedaliera di Padova, Via Roma, 67, Padova, 35128
2003035 Istituto Giannina Gaslini, Largo G. Gaslini, 5, Genova, Italy 16100
2003036 Doctors Hospital at Renaissanc, 2609 Michael Angelo Dr, Edinburg, TX 78539
2003038 KIDZ Pediatric Multispecialty, 6280 Sunset Drive, Suite 607, South Miami, FL 33143
2003060 UNESP, Rua Professor Doutor Armando Alves s/n, UNESP Botucatu Rubião Júnior, Botucatu, Sao Paulo 18618-970
2003062 CHU CAEN - CHR, Clémenceau, Avenue Georges Clemenceau, Caen Cedex, Calvados 14033
2003063 Tolna Megyei Balassa János Kh, Béri Balogh Ádám u 5-7, Szekszárd, 7100
2003065 TO - A.O. Città della Salute, Azienda Ospedaliera Città della Salute e della Scienza di Torino, Corso Spezia 60, Torino, Torino 10126
2003066 Christchurch Hospital, 2 Riccarton Ave, Paediatric Department, Christchurch, 8011
2003067 Cincinnati Children's Hospital, T Building Room T11, 240 Albert Sabin Way, Cincinnati, OH 45229
2003068 Children's Hospital of Pittsburgh, 4401 Penn Avenue 5th Floor Main Hospital, Pittsburgh, PA 15224
2003069 Augusta University, Georgia Regents University, 1120 15th Street, IBA-7411, Augusta, GA 30912
2003078 DaVita Clinical Trials, 6340 Barnes Rd, Colorado Springs, CO 80922
2003079 Advanced Research Center, 1020 S Anaheim Blvd, Suite 316, Anaheim, CA 92805
2003092 Univ of No Texas Health Sci, Office of Clinical Trials, 3500 Camp Bowie Blvd, Fort Worth, TX 76107
2003093 University of Missouri, 1 Hospital Dr, Columbia, MO 65212
2003094 CIEC, Manzano, 343 - Oficina 410, Recoleta, Santiago, 8420383
2003095 Hospital Universitario Clinico, Professor Martin Lagos S/N, Hospital Clinico San Carlos, Madrid, Madrid 28040
2003118 Long Beach Memorial Medical Ct, 2801 Atlantic Ave, Pediatric Infectious Diseases, Long Beach, CA 90806
2003124 Center for Clinical Trials, 16660 Paramount Blvd, Suite 301, Paramount, CA 90723
2003125 Ohio Pediatric Research Association, 7200 Poe Ave, Suite 200, Dayton, OH 45414
2003167 Woburn Pediatric Associates, 7 Alfred Street, Baldwin Park II, Woburn, MA 01801
2003257 Hospital de Niños y Cunas, Limache 1667, Viña del Mar, 2520594
2003273 Universitair Ziekenhuis, Gent, De Pintelaan 185, Gent, 9000
2003274 CHU Ambroise Paré, Boulevard Kennedy 2, Mons, 7000
2003276 Maychin Dom Hospital, ul Zdrave 2, 1431 Sofia Center, Sofia, 1431
2003280 McGill University Health Centr, 1001 Decarie Blvd, Montreal Childrens Hospital, Montreal, QC H4A 3J1
2003282 Tallinn Children's Hospital, Tervise 28, Tallinn, 13419
2003283 Tartu University Hospital, Children's Clinic, 6 N Lunini St, Tartu, 51014
2003286 Tampereen rokotetutkimusklinik, Pinninkatu 47, 1st Floor, Tampere, 33100
2003287 Akdeniz University Hospital, Pediatrics Hematology and Oncology Department, Block H, Ground Level, Antalya, 07070
2003288 Hacettepe Univ Medical Faculty, Ihsan Dogramaci Children's Hospital, Department of Pediatrics Division of Neonatalogy, Ankara, Altindag 06100
2003289 Izmir Dr Behcet Uz, Child Diseases & Surgery Training & Research Hospital, Intensive Care Clinic, Izmir, Konak / Izmir 35210
2003290 Ege University Medical Faculty, Department of Pediatrics and Neonatology, Izmir, Bornova 35100
2003291 Cukurova Univ Medical Facility, Balcali Hospital, Department of Pediatrics Health and Diseases, Division of Neonatology, Adana, Saricam 1260
2003293 Turun rokotetutkimusklinikka, Lemminkäisenkatu 14-18 B, 4th Floor, Turku, 20520
2003301 Hopital Clocheville, Pediatric and Neonatal Resuscitation Service, 49 Boulevard Beranger, Tours CEDEX 9, Indre et Loire 37044
2003302 SzSzB Megyei Korhazak es Egyet, Josa Andras Oktatokorhaz Gyermekosztaly, Szent Istvan u 68, Nyireyghaza, 4400
2003303 Central Hosp of Bekes County, Karpat Road 11, Gyula, 5700
2003304 Csolnoky Ferenc Kórház, Csecsemo and the Children's Health Center, Kórház u 1, Veszprém, 8200
2003305 Vidzemes Hospital, Jumaras 195, Valmiera, Latvia 4200
2003307 Jekabpils Regional Hospital, Pormala Street 125, Jekabpils, LV- 5201
2003310 Hospital of Lithuanian, Eiveniu g 2, Kaunas, 50161
2003311 Hospital Universitario Virgen, Avenida de las Fuerzas Armadas s/n, Granada, Granada 18014
2003312 University Hospital of Alicante, Neonatology Department, Pintor Baeza 11, Building D, 4th Floor, Alicante, Alicante 03010
2003314 Södersjukhuset, Sachsska barn-och Ungdomssjukhuset, Sjukhusbacken 10, Stockholm, 118 83
2003315 Karolinska University Hospital, Karolinska Universitetssjukhuset, PO Sjuka Nyfodda Barn, Stockholm, 171 76
2003320 Royal Alexandra Childrens Hospital, Eastern Road, Brighton, BN2 5BE
2003326 Kocaeli Univ Medical Faculty, Clinical Trials Division, Level 1, Umuttepe Campus, Kocaeli, 41380
2003328 Univ of Nebraska Med Center, Department of Pediatrics, 982162 Nebraska Medical Center, Omaha, NE 68198
2003329 Wee Care Pediatrics, 1792 W 1700 South, Suite 102, Syracuse, UT 84075
2003330 Arnold Palmer Hosp Specialty, 83 W Columbia Street, Orlando, FL 32806
2003331 Cohen Children's Medical Center, 269-01 76 Ave. New Hyde Park, New Hyde Park, NY 11040
2003333 Children's Hospital Los Angeles, 4650 Sunset Blvd, Mail Stop 51, Los Angeles, CA 90027
2003334 Stony Brook University Hospital, The Research Foundation of SUNY, 101 Nicolls Road, Stony Brook, NY 11794
2003338 Palmetto Pediatrics, PA, 2781 Tricom St, North Charleston, SC 29406

2003339 Marshall Health, 1600 Medical Center Drive, Suite 3500, Huntington, WV 25701
2003340 Center for Clinical Trials, 1535 W Merced Ave, Suite 208, West Covina, CA 91790
2003341 NYU Winthrop Hospital, 120 Mineola Boulevard Suite 210, Mineola, NY 11501
2003342 Aventiv Research Inc, 99 N Brice Road, Suite 260, Columbus, OH 43231
2003343 Sharp Healthcare, Mary Birch Hospital For Women and Newborns, 3003 Health Center Drive, San Diego, CA 92123
2003346 Alabama Clinical Therapeutics, Birmingham Pediatrics Research, 806 St Vincent's Drive, Suite 615, Birmingham, AL 35205
2003348 Capitol Pediatrics & Adolescents, 3801 Computer Dr, Raleigh, NC 27609
2003349 Meridian Clinical Research, 3319 N 107th St, Omaha, NE 68134
2003352 University of Tennessee, CFRI, Department of Pediatrics, 50 N Dunlap St, Room 400R, Memphis, TN 38103
2003353 Utah Valley Pediatrics, 716 W 800 North, Suite 300, Orem, UT 84057
2003354 Sanford Research/USD-Sioux Falls, 1018 West 18th St, Sioux Falls, SD 57104
2003355 Cyn3rgy Research, 24850 SE Stark Street, Suite 180, Gresham, OR 97030
2003356 Univ of Miss Medical Center, 2500 N State St, Jackson, MS 39216
2003358 Medical Univ of South Carolina, 135 Rutledge Avenue, MSC 550, Charleston, SC 29425
2003359 Metro Health System, 2500 MetroHealth Drive, Cleveland, OH 44109
2003394 Tanner Clinic, 2121 N 1700 West, Layton, UT 84041
2003395 Blue Ridge Pediatric & Adolescends, 579 Greenway Rd, Suite 200, Boone, NC 28607
2003402 Midwest Children's Health, 3262 Salt Creek Circle, Lincoln, NE 68504
2003405 Memorial Hosp of South Bend, 615 N Michigan Street, South Bend, IN 46601
2003406 Connecticut Childrens Medical, 282 Washington St, Suite 4B, Hartford, CT 06106
2003407 Allegheny Health Network, , AHN Health & Wellness Pavilion, 4247 West Ridge Road, Erie, PA 16506
2003408 Pauls Stradins Clinical Univ H, Pilsonu Street 13, Riga, LV1002
2003410 InMedica, JSC, Baltu pr 7A, Kaunas, 48259
2003411 Hospital Universitario Dr Pese, Servicio de Pediatria, Avda Gaspar Aguilar 90, Valencia, 46017
2003412 Univ Hosp Madrid Monteprincipe, Teaching Building CEU, Second Floor UCEC Office, Avenida Monteprincipe 25, Boadilla del Monte, Madrid 28660
2003413 Hosp Universitario Reina Sofia, Avda Menendez Pidal s/n, Servicio de Pediatria, Cordoba, 14004
2003414 Hosp Univ San Juan de Alicant, Ctra Alicante - Valencia s/n, San Juan de Alicante, 03550
2003415 Hospital Univ Arnau de Vilanov, Avinguda Rovira Roure 80, Lleida, 25198
2003442 DCOL Ctr for Clinical Reseach, 707 Hollybrook Drive, Suite 501, Longview, TX 75604
2003444 Road Runner Research, 4374 Lockhill-Selma Rd, Suite 108, San Antonio, TX 78249
2003447 Univ of Wisconsin- Madison, 600 Highland Avenue, Madison, WI 53792
2003448 University of Oklahoma, 800 NE 10th Street, Suite 2100, Oklahoma City, OK 73104

MELODY study sites
Site # Centre address
2004132 9 de Julio 279, San Miguel de Tucuman
2004648 Hospital del Niño Jesús [Niño Jesús Hospital], Pasaje Hungria 750, San Miguel de Tucuman
2004327 Level 4,246 Clayton Road,Monash Children’s Hospital,Nursing & Medical Administration, Clayton
2004373 Office 5 East, 15 Hospital Avenue, NEDLANDS
2004249 Auenbruggerplatz 34/2 Medizinische Universität Graz, Univ.-Klinik für Kinder- und Jugendheilkunde, Graz
2004298 Waehringer Guertel 18 - 20, Vienna
2004231 Laarbeeklaan 101, Bruxelles
2004270 Rue Haute 322, Bruxelles
2004320 Corneel Heymanslaan 10 Poli Neurologie, entrance 12, route 1525, Gent
2004398 Route de Lennik 808., Bruxelles
2004629 Ruddershove 10 Campus Sint-Jan, Brugge
2004303 17 Stoev str, Plovdiv
2004324 2, Nezavisimost Str., Ruse
2004325 66, Peshtersko Shose Str. Second Clinical Base, Plovdiv
2004339 91, Gen. Vladimir Vazov Str. Second Clinical Base, Pleven
2004343 2, Sirma Voyvoda Str., Montana
2004399 51B, Nikola Vapcarov Blvd., Sofia
2004401 63, Sergey Rumiantzev Str., Veliko Tarnovo
2004639 15, Bolnichna Str., Pazardzhik
2004365 14770 boul. Pierrefonds Suite 204, Pierrefonds
2004623 K-3202-4480 Oak Street CF Lab Research Room 2C75, Vancouver
2004667 28 Oki Drive NW, Calgary
2004043 Esperanza 2150 San Ramon, Santiago
2004233 Carrera 72 A # 78 B 50, Medellin
2004295 Carrera 9 No. 7-33 / Calle 5B5 No.37 Bis-28, Cali
2004304 Carrera 48 No 32-102, Medellin
2004372 Carrera 4 Este N° 24-65, Chia
2004400 Calle 30 Via el Aeropuerto al lado del parque Muvdi, Soledad
2004669 Cra 3 # 21 - 44, Monteria
2004044 Husova 2624, Havlickuv Brod
2004045 Paldiski mnt 68A, Tallinn
2004046 Vee 6, Paide

2004047 N. Lunini 6, Tartu
2004099 Tervise 28, Tallinn
2004273 Paldiski mnt 68a, Tallinn
2004274 Narva mnt 7, Tallinn
2004312 Pinninkatu 47, 1. krs, Tampere
2004313 Lemminkäisenkatu 14-18 B, 4. krs, Turku
2004611 Mannilantie 44, 2.krs, Järvenpää
2004644 Kiviharjunlenkki 6, Oulu
2004741 Lemminkäisenkatu 14-18 B, 4. krs, Turku
2004742 Kauppatori 1-3, 2. krs, Kauppakeskus Torikeskus, Seinäjoki
2004743 Rantakatu 7, Maximin liikekeskus 2. krs, Kokkola
2004887 Piispansilta 11 Ison Omenan toimistotorni, 7. krs, Espoo
2004896 Vuorikatu 18, 3. krs, Helsinki
2004100 5eme Secteur du Val-de-Marne 40 avenue de Verdun, Creteil cedex
2004232 59 Boulevard Pinel, Bron
2004374 2 Avenue Foch, Brest
2004378 1 Rond point du Professeur Christian Cabrol RDC HALL 2, Amiens
2004654 Avenue Cote de Nacre, Caen
2004676 Place Amelie Raba Leon, Bordeaux
2004178 Collinistrasse 11, Mannheim
2004182 Achenweg 1, Schönau am Königssee
2004185 Liebigstrasse 20a, Leipzig
2004359 Welschgasse 39, Frankenthal
2004626 Itzchak Rager Boulevard P.O.Box 151, Beer-Sheva
2004672 16 Divrei Chaim St., Netanya
2004396 via Tommaso Gargollo 24 Largo Agostino Gemelli, 8, Roma
2004632 Asakura-machi 389-1, Maebashi-shi
2004660 Yonegahamadori 1-16, Yokosuka-shi
2004668 Naniwa-ku Nippombashi 5-16-15, Osaka-shi
2004670 Kita-ku Tamasu 1711-1, Okayama-shi
2004671 Kawasaki-ku Shinkawadori 12-1, Kawasaki-shi
2004687 Zao-cho 5-23-1, Fukuyama-shi
2004688 Toyohira-ku Hiragishi 1jo 6-3-40, Sapporo-shi
2005029 Wadanaka-cho Funabashi 7-1, Fukui-shi
2005030 Kita-ku Kitanagaseomote-machi 3-20-1, Okayama-shi
2005031 Naka-ku Moto-machi 7-33, Hiroshima-shi
2005032 Wakasato 5-22-1, Nagano-shi
2005033 Kosobe-cho 1-3-13, Takatsuki-shi
2005034 Tsunatorihon-machi 12-1, Isesaki-shi
2005036 Aoi-ku Kitaban-cho 23, Shizuoka-shi
2005037 Naka-ku Senda-machi 1-9-6, Hiroshima-shi
2005038 Senyu-cho 2-1-1, Zentsuji-shi
2005039 Minami-ku Hakusui-cho 8, Nagoya-shi
2005049 Nakagawa-ku Shonen-cho 4-66, Nagoya-shi
2004769 50 Yonsei-ro, Seodaemun-gu, Seoul
2004798 27, Inhang-ro, Jung-gu, Incheon
2004199 Pormala street 125, Jekabpils
2004202 Pilsonu street 13 Pauls Stradins University Hospital, Riga
2004302 L. Paegles street 9, Valmiera
2004331 Jumaras street 195, Valmiera
2004380 Vienibas street 45, Riga
2004204 Eiveniu g. 2, Kaunas
2004277 Baltu pr. 7A, Kaunas
2004710 Baltu pr. 7, Kaunas
2004404 Dr Marquez 162 Col Doctores Del Cuauhtemoc, Mexico
2004048 Clinical Trials Unit, Level 8 - CSB Building,,Wellington Hospital, Riddiford Street,,Newtown,Wellington
2004105 Level 4, 264 Antigua Street, Christchurch 8011 New Zealand, Christchurch
2004335 2 Park Road Auckland City Hospital, Grafton
2004405 Middlemore Clinical Trials 100 Hospital Road, Papatoetoe
2004867 Avenida 3ra Oeste con Calle D y E Norte,50 metros de Mini Baru, Doleguita David, David
2004868 Avenida México, Calle 33, local #4, Calidonia, Panama
2004869 Calle 69 Oeste, Casa No. 76 en frente de Nestle. Consultorio No. 1, Panama
2004870 Av. Las Americas y calle El Carmen Chorrera, Panama
2004871 Vía Jose Agustin Arango - Parque Lefevre PH Plaza Carolina, Local #10, Panama City
2004872 Urbanizacion Nuevo Tocumen, calle 1 y 2 edificio plaza nuevo tocumen locales A2 A3 Y A5, Cuidad de Panama
2004049 ul. Waclawa Jawoszka 3, Leczna
2004208 ul. Pod Fortem 2F/7, Krakow
2004305 ul. Szafrana 5D/U2, U4, U5, Kraków
2004350 ul. Mariana Smoluchowskiego 17, Gdansk

2004371 ul. Rafała Wojaczka 3C, Wroclaw
2004381 ul. Stefana Batorego 18-22, Torun
2004674 ul. Raclawicka 105/1B, Wroclaw
2004106 10, Tovarischeskiy prosp., Saint-Petersburg
2004212 Vosstaniya str., 8. Lit.A, Saint-Petersurg
2004214 10, Sovetskoy Armii Str. Lit. A, Perm
2004336 3 Detskiy per, St.Petersburg
2004034 St Augustine Medical Centre, Suite 111 JB Marks Road, Durban
2004036 Klipfontein Road Rondebosch, Cape Town
2004039 Ward J8, Francie van Zijl Drive, Parow Valley, Stellenbosch University, Cape Town
2004108 Unit U3-U7, The Enterprise Building, The Innovation Hub, 6 Mark Shuttleworth Street, Pretoria
2004109 11th Floor West Wing, New Nurses Residence Chris Hani Road, Soweto
2004110 8498 Khubone Drive, Mamelodi West Pretoria
2004111 2nd Floor Off St Johns Road, Wynberg, Cape Town
2004216 B Block, 1st Floor,Fuel Road & Oudtshoorn Street, Coronationville, Johannesburg
2004217 Ward 477, 7th floor, Green block, Jubilee Road, Parktown, Johannesburg
2004712 1 De Lange street Bellville, Cape Town
2004033 Ctra. Alicante - Valencia, s/n Neurologia, San Juan de Alicante
2004113 C/ Pere i Pons, 1 Pediatría, Sant Cugat del Valles
2004114 Av. Imperio Argentina, 1 Digestivo, Malaga
2004115 Avda. Fuerzas Armadas, s/n Servicio de Pediatria, Granada
2004218 Travesia da Choupana s/n Pediatria, Santiago de Compostela
2004219 Avinguda Rovira Roure, 80, Lleida
2004311 Ofra s/n Ctra. La Cuesta-Taco, San Cristobal De La Laguna
2004333 Avda. de Orellana, s/n Servicio Pediatria, Leganes
2004344 c/ Diego de Velazquez 1 Servicio Pediatria, Pozuelo de Alarcon
2004363 Avda. Pintor Baeza, 12 Pediatria, Alicante
2004369 Avda. Gaspar Aguilar, 90, Valencia
2004382 Avenida Benicassim s/n Servicio Pediatria, Castellon de la Plana
2004385 Avda. Menendez Pidal, s/n Servicio de Pediatria, Cordoba
2004406 Profesor Martin Lagos s/n Pediatria, Madrid
2004407 Avda. Poeta Muñoz Rojas, S/n Servicio de Farmacia, Antequera
2004675 Avenida de Carlos V, 70, Mostoles
2004116 Sachsska barn och ungdomssjukhuset, Södersjukhuset, Sjukhusbacken 10, SE-118 83 Stockholm
2004402 Universitessjukhuset, Allergicentrum Brigadgatan 14, plan 3, SE-587 58 Linköping
2004221 Cukurova Uni. Tip Fak. Yenidogan BD,Balcali Hastanesi,Balcali, Adana
2004222 Ege Universitesi Tip Fakultesi Cocuk Sagligi ve Hastaliklari ABD Bornova, Izmir
2004223 Hacettepe Universitesi Tip Fakultesi, Acil Tip Anabilimdali, Sihhiye, Ankara
2004224 Kocaeli Universitesi Tip Fak.,Umuttepe Merkez Yerleskesi, Onkoloji Anabilimdali,Kocaeli
2004117 4, Bukovynska St., Chernivtsi
2004229 108, Khmelnytske Shose Str., Vinnytsia
2004294 8, Platona Maiborody St., Kyiv
2004633 29, V.Antonovycha Str., Dnipro
2004383 Eaton Road, Liverpool
2004384 Room No.160, Ground Floor, Jenner Wing St George's, University of London, London
2004023 579 Greenway Road Suite 200, Boone
2004025 2781 Tricom Street, North Charleston
2004026 1200 Everett Drive,Rm 5E219,The Children's Hospital at OU Medicine, Everett Tower Pharmacy, Oklahoma City
2004027 5323 S. McColl Road, Edinburg
2004028 211 11th Street NE, Charlottesville
2004030 5612 NW 43rd Street, Gainesvlle
2004031 105 West Stone Dr 3rd Floor, Suite 3B, Kingsport
2004032 4374 Lockhill-Selma Rd Ste 108, San Antonio
2004118 5657 South Himalaya Street Suite #100, Centennial
2004236 806 St. Vincent's Drive Suite 615, Birmingham
2004237 4247 West Ridge Road Suite 105, Erie
2004238 225 E. Chicago Ave., Box 107, Chicago
2004239 1433 W. Merced Ave Suite 102, West Covina
2004240 16660 Paramount Blvd #301, Paramount
2004241 3333 Burnett Avenue, Cincinnati
2004243 6340 Barnes Road, Colorado Springs
2004255 7200 Poe Ave Suite 200, Dayton
2004258 8535 Florence Ave. Suite 201, Downey
2004259 750 E. Adams Street, Suite 5400, Syracuse
2004260 2121 North 1700 West, Layton
2004261 2500 N State Street, Jackson
2004263 1000 East Broad Street Room 5-253, Richmond
2004268 4040 Finn Way Ste 310, Lexington
2004279 1100 Salem Ave., Dayton

2004280 707 Hollybrook Drive, Suite 501, Longview
2004281 2500 MetroHealth Drive, 4th floor towers, room 468, Cleveland
2004291 1 Medical Center Dr. WVU Medicine Physicians Office Center, Morgantown
2004292 201 South 5th Street Suite 102, Bardstown
2004293 2400 NW 54th Street, Miami
2004314 650 East 4500 South Suite 100, Salt Lake City
2004315 2084 North 1700 West, Suite A, Layton
2004316 5682 South 3500 West Suite A, Roy
2004319 10905 Memorial Hermann Dr. Suite 109, Pearland
2004323 1600 W. 22nd Street, Sioux Falls
2004340 1717 East Bert Kouns Industrial Loop, Shreveport
2004376 Clinical Research Center One Hospital Drive Room N508, Columbia
2004386 120 Mineola Blvd Suite 210, Mineola
2004389 1330 Cedar Lane Building B Suite 900, TULLAHOMA
2004391 8200 Dodge Street IHW 5th Floor, Omaha
2004394 1240 E 100 S Ste 14, St. George
2004409 3414 Fifth Avenue CHOB 3rd Floor, Pittsburgh
2004613 9480 Huebner Road Suite 400, San Antonio
2004614 1690 West Baker Road Suite B, Baytown
2004615 655 Euclid Avenue, Suite 205, National City
2004618 425 W 51st Place, Hialeah
2004624 4440 W 95th St, Oak Lawn
2004634 1120 15th St, CJ-1117, Augusta
2004652 705 Riley Hospital Drive, ROC 4270, Indianapolis
2004657 3262 Salt Creek Circle, Lincoln
2004658 700 Childrens Drive Suite T6B, Columbus
2004662 50 North Dunlap St. Room No. 401R, Memphis
2004664 4800 Sandpoint Way NE, Seattle
2004677 3556 West 9800 South, Suite 101, South Jordan
2004678 1000 N Oak Ave, Marshfield
2004680 2785 Gulf Freeway South Suite 200, League City
2004690 3383 N. Mana Ct. St 101, Fayetteville
2004699 27721 Tomball Parkway Suite 100, Tomball
2004700 5950 University Ave., West Des Moines
2004702 2054 S Green Rd, Cleveland
2004708 2015 Uppergate Drive, Room 542, Atlanta
2004746 4200 Wisconsin Ave NW Suite 200, Washington
2004747 No 3 Cottonwood Street, Shiprock
2004748 No 3 Cottonwood Street, Shiprock
2004749 No 3 Cottonwood Street, Shiprock
2005605 12221 Merit Drive Suite 350, Dallas
2005606 2660 10th Ave. S., Building 1 Suite 735, Birmingham

Limitations of recruitment included: 1) infants from the phase 2b study were less diverse geographically due to restrictions related to future use consent laws for biosamples in several countries; 2) the COVID-19 pandemic created an off-cycle RSV season in 2020–2021 where lockdowns, masking, and social distancing changed the incidence and prevalence of RSV; 3) infants could have been exposed to RSV prior to randomization if they were aged >6 months.

Ethics oversight

The IRB/IEC responsible for each site reviewed and approved the final study protocols, including the final version of the informed consent form and other written information and/or materials provided to the subjects. The IRB/IEC also approved all advertising used to recruit subjects for the study. The investigator was responsible for submitting the documents to the applicable IRB/IEC, and distributing them to the study site staff.
Site Number Name/Address of IRB/IEC
Phase 2b
2002923 Pharma Ethics 123 Amcor Road Lyttelton Manor Centurion Pretoria Gauteng
2003359 MetroHealth Medical Center IRB 2500 MetroHealth Dr. Rammelkamp Bldg. Room 103 Cleveland Ohio
2002934 CEP Investiga - Instituto de Pesquisas Avenida Romeu Tortima, 739 - Cidade Universitária Campinas Sao Paulo
2002970, 2003091, 2003395, 2003007, 2003356, 2003405, 2003355, 2003354, 2003004, 2003353, 2002971, 2003124, 2003350, 2003394, 2003092, 2003348, 2003078, 2002974, 2003036, 2003346, 2003340, 2003441, 2003167, 2003338, 2003337, 2003442, 2003068, 2003038, 2003342, 2003335, 2003399, 2003086, 2003444, 2003400, 2003332, 2002976, 2003402, 2003347, 2003403, 2003125, 2003329, 2003336, 2003079, 2003401, 2003407 Copernicus Group IRB 5000 CentreGreen Way Suite 200 Cary North Carolina Adams, Gregory
2002935 CEP da Universidade Federal de Minas Gerais Avenida Presidente Antonio Carlos 6627 Unidade Administrativa II Belo Horizonte Minas Gerais Andrade
2002947 CEIC de Galicia C/ San Lázaro, s/n Secretaria Xeral. Conselleria de SanidadeDirección Santiago de Compostela La Coruña Ares
2002948 CEIC de Galicia C/ San Lázaro, s/n Secretaria Xeral. Conselleria de SanidadeDirección Santiago de Compostela La Coruña Arimany Montaña,
2003358 Medical University of South Carolina IRB 19 Hagood Avenue 6th floor, Suite 601 Charleston South Carolina
2002918 Wits Health Consortium 31 Princess of Wales Terrace Parktown Johannesburg Gauteng

2002998 Comite Etico Cientifico del Servicio de Salud Metropolitano Sur Santa Rosa 3453, Piso 1 San Miguel Santiago
2003034 CESC della Provincia di Padova Presso Azienda Ospedaliera di Padova_Via Giustiniani 1 Padova
2002939 CEP da Faculdade de Ciências Médicas e da Saúde de Juiz de Fora SUPREMA/MG Alameda Salvaterra, 200 Bairro Salvaterra Juiz de Fora Minas Gerais Bastos
2003060 CEP da Faculdade de Medicina de Botucatu - UNESP/SP Distrito de Rubião Junior Botucatu Sao Paulo
2003000 Comitato Etico per la Sperimentazione Clinica delle Provincie di Verona e Rovigo P.le Stefani, 1 Verona
2002919 Pharma Ethics 123 Amcor Road Lyttelton Manor Centurion Pretoria
2002910 Monash Health Human Research Ethics Committee (RGO) Level 2, I Block Clayton Victoria
2002953 Comité Etico Científico Servicio de Salud Valdivia Maipú 550, oficina 307 Valdivia
2002920 University of Stellenbosch Ethics Committee Faculty of Health Sciences Francie van Zijl Drive Tygerberg Cape Town Western Cape
2002956 Comité Ético Científico Servicio de Salud Metropolitano Central Victoria Subercaseaux 381, piso 4 Santiago
2003352 UTHSC IRB Office 910 Madison Suite 600 Memphis Tennessee
2003118 Memorial Health Services Research Council 2801 Atlantic Avenue Attn Research Administration Long Beach California
2002972 SUNY IRB 750 East Adams Street CWB 218G Syracuse New York
2003277 McGill University Health Center-Research Ethics Board 2155 Guy Street 2nd Floor, Room 231 Montreal Quebec
2002921 Pharma Ethics 123 Amcor Road Lyttelton Manor Centurion Pretoria Gauteng
2002973, 2003061, 2003069, 2003093, 2003331, 2003447 WIRB 1019 39th Avenue SE Suite 120 Puyallup Washington
2002905 Comité de Ética en Investigación Científica. Hospital Pediátrico Dr. Humberto Notti Bandera de Los Andes 2603 Villa Nueva Guaymallén Mendoza
2002967 R&D University Hospital Southampton NHS Foundation Trust Tremona Road, Level E, Laboratory & Pathology Block, SCBR - MP 138 Southampton Hampshire
2003320 R&D - Brighton and Sussex University Hospitals Royal Sussex County Hospital Level 5 Thomas Kemp Tower Eastern Road Brighton East Sussex
2003065 Azienda Ospedaliera Città della Salute e della Scienza di Torino Corso Bramante 88/90. Torino
2002940 Comitê de Ética em Pesquisa em Seres Humanos do Instituto de Medicina Integral Professor Fernando F Rua dos Coelhos, 300 - Boa Vista Recife Pernambuco Gomes
2002922 University of Cape Town HREC Faculty of Health Sciences Research EC E52-24 Old Main Building Groote Schuur Hospital, Observatory Cape Town Western Cape
2002941 CEP da Universidade Luterana do Brasil - ULBRA Farroupilha, 8001 - Prédio 14 - Sala 224 Bairro São José Canoas Rio Grande do Sul
2003319 R&D - Alder Hey Children's NHS Foundation Trust Eaton Road Liverpool Merseyside
2002968 R&D South West London and St George's Mental Health NHS Trust Department of Mental Health, St George's, University of London, 6th Floor, Hunter Wing, Cranmer Terrace London Greater London
2002924 Eticka komise IKEM a FTNsP Videnska 800 Praha 4 - Krc
2003343 Sharp Healthcare IRB 7930 Frost St Suite 300 San Diego California
2003341 Winthrop-University Hospital IRB 222 Station Plaza North Suite 521 Mineola New York
2002943 Comitê de Ética em Pesquisa em Seres Humanos do Hospital Pequeno Príncipe Rua Desembargador Motta, 1070 6º andar, sala do NUPE Curitiba Paraná
2003339 Marshall University Office of Research Integrity One John Marshall Drive Huntington West Virginia
2002937 Wits Health Consortium 31 Princess of Wales Terrace Parktown Johannesburg Gauteng
2002950 CEIC de Galicia C/ San Lázaro, s/n Secretaria Xeral. Conselleria de SanidadeDirección Santiago de Compostela La Coruña Martinon
2002944 CEP da Universidade de Passo Fundo/RS Universidade de Passo Fundo - BR 285, Bairro São José Passo Fundo Rio Grande do Sul
2003011 Ann & Robert H. Lurie Children's Hospital of Chicago Institutional Review Board 225 E. Chicago Avenue Box 59 Chicago Illinois
2003334, 2002975 Chesapeake IRB 7063 Columbia Gateway Drive Suite 110 Columbia Maryland
2003067 Cincinnati Children's Hospital Medical Center IRB 3333 Burnet Ave. MLC 5020 Cincinnati Ohio
2002954 Comité Ético-Científico Servicio de Salud Metropolitano Sur Oriente Av Concha y Toro 3459 Puente Alto Santiago
2003333 Childrens Hospital of Los Angeles-Committee on Clinical Investigations IRB 4650 Sunset Blvd Mail Stop #23 Dr. Andreas Reiff Los Angeles California
2003280 McGill University Health Center-Research Ethics Board 2155 Guy Street 2nd Floor, Room 231 Montreal Quebec
2003005 University of Texas at San Antonio IRB One UTSA Circle MS 4.01.82 San Antonio Texas
2002951 CEIC de Galicia C/ San Lázaro, s/n Secretaria Xeral. Conselleria de SanidadeDirección Santiago de Compostela La Coruña
2002911 Royal Children's Health Services Human Research Ethics Committee (RGO) 50 Flemington Road Parkville Victoria
2002938 Pharma Ethics 123 Amcor Road Lyttelton Manor Centurion Pretoria Gauteng
2003257 Comité Ético-Científico Servicio de Salud Viña del Mar-Quillota Calle Limache #1307 Esquina Peñablanca 2º Piso Viña del Mar Quilodran
2003035 Comitato Etico Regionale della Liguria Largo Rosanna Benzi 10 Farmacia Ospedaliera Genova
2002912 Princess Margaret Hospital for Children Ethics Committee Princess Margaret Hospital Entrance No 6, Hamilton Street Subiaco Western Australia
2003330 Arnold Palmer Medical Center Institutional Review Board 1401 Kuhl Avenue MP #21 Research Department Orlando Florida
2003328 University of Nebraska Medical Center IRB 987830 Nebaska Medical Center Omaha Nebraska
2002969 R&D - CRN Thames Valley and South Midlands 1st Floor, Manor House The John Radcliffe Hospital, Headley Way Headington Oxford Oxfordshire
2002926 Eticka komise Ustav pro peci o matku a dite Podolske nabrezi 157/36 Praha 4 - Podoli

2002909 Comité Hospitalario de Etica Necochea 675 Bahia Blanca Buenos Aires
2003274 Comité d'Ethique du CHU Ambroise Paré Boulevard Kennedy 2 Mons Van
2002955 Comité de Ética de Investigación en Seres Humanos Av. Independencia 1027, Independencia Santiago Vargas
2003009 Creighton University IRB 2500 California Plaza IRB-Biomedical Omaha Nebraska
2002966 R&D University Hospitals Bristol NHS Foundation Trust Education & Research Centre Level 3 Upper Maudlin Street Bristol Avon
2002999 Comite Etico Cientifico del Servicio de Salud Metropolitano Sur Santa Rosa 3453, Piso 1 San Miguel Santiago Villena
2002927 Eticka komise Nemocnice Havlickuv Brod Husova 2624 Havlickuv Brod Weberova,
2003448 Oklahoma University Health Sciences Center 1105 North Stonewall Avenue Oklahoma City Oklahoma
2003406 Connecticut Children's Medical Center IRB 282 Washington Street. Suite 2 K. Hartford Connecticut
2002946 University of Cape Town HREC Faculty of Health Sciences Research EC E52-24 Old Main Building Groote Schuur Hospital, Observatory Cape Town Western Cape
MELODY
2004023, 2004025, 2004027, 2004028, 2004030, 2004031, 2004032, 2004118, 2004236, 2004237, 2004239, 2004240, 2004243, 2004253, 2004255, 2004256, 2004258, 2004259, 2004260, 2004261, 2004263, 2004264, 2004267, 2004268, 2004278, 2004279, 2004280, 2004291, 2004292, 2004293, 2004314, 2004315, 2004316, 2004319, 2004323, 2004340, 2004345, 2004376, 2004386, 2004389, 2004394, 2004409, 2004613, 2004614, 2004615, 2004618, 2004624, 2004634, 2004650, 2004652, 2004656, 2004657, 2004664, 2004677, 2004679, 2004680, 2004690, 2004697, 2004699, 2004700, 2004702, 2004746, 2004873, 2005604, 2005605, 2005606 WCG IRB, 212 Carnegie Center, Suite 301, Princeton, NJ 08540, USA
2004026 The University of Oklahoma, Institutional Review Board for the Protection of Human Subjects, 1105N. Stone wall Avenue, Oklahoma City, OK73117(FWA 007961)
2004029 Nemours Office of Human Subjects Protection, Nemours/Alfred I. duPont Hospital for Children, 1600 Rockland Road, Wilmington, DE 19803
2004036 University of Cape Town Human Research Ethics Committee, DEPARTMENT OF PAEDIATRICS AND CHILD HEALTH, RED CROSS WAR MEMORIAL CHILDREN'S HOSPITAL, KLIPFONTEIN ROAD, RONDEBOSCH, 7700
2004039 Stellenbosch University Human Research Ethics Committee, Stellenbosch University, Private Bag X1, Matieland, 7602, Stellenbosch, South Africa
2004043 Servicio De Salud Metropolitano Sur Oriente Comite Etico-Cientifico, Av. Concha y Toro 3459 – Paradero 30, Vic. Mackenna
2004098 UNIVERSIDAD DE CHILE [University of Chile] – FACULTAD DE MEDICINA, HUMAN RESEARCH ETHICS COMMITTEE, Av. Libertador Bernardo O'Higgins 1058, Santiago de Chile
2004103 Comitato Etico per la Sperimentazione Clinica delle Provincie di Verona e Rovigo,P.le Stefani, 1, Verona,37126
2004111 1 Military Hospital Human Research Ethics Committee, Department of Neurology Private bag X 1026 Thaba Tswane 0143
2004117 Dept of health of Chernivtsi city council, Communal Medical Institution City Clinical Childrens' Hospital, 4 Bukovynska St, Chernivtsi, 58001
2004132 Independent Ethics Committee for Clinical Pharmacology Trials, Drug and Pharmacology Studies Foundation, LA FUNDACIÓN DE ESTUDIOS FARMACOLOGICOS Y DE MEDICAMENTOS, Pte. J. E. Uriburu 774 1º Piso Ciudad Autónoma de Buenos Aires (C1027AAP), Argentina
2004178 Landesärztekammer Baden-Württemberg, Ethik-Kommission, Liebknechtstr. 33, 70565 Stuttgart
2004182 Ethik-Kommission der Bayerischen Landesärztekammer,
Mühlbaurstr.16, D-81677 München
2004185 Ethik-Kommission an der Medizinischen Fakultaet der Universitaet Leipzig,Kaethe-Kollwitz-Strasse 82,Haus: Karl-Sudhoff-Institut Leipzig,04109
2004222, Ege University Ethics Committee,Ege Universitesi Tip Fakultesi,Klinik Arastirmalar Etik Kurulu Izmir,35100
2004227 Ministy of Health of Ukrain, Communal Non-Commercial enterprise Saint Zinaida Children's Clinical Hospital of Sumy City Council, 28 Troiiska st, Sumy, 40022
2004229 Vinnytsia regional Children's Clinical Hospital, 108 Khmelnytske shose st, Vinnytsia, 21000. Medical Ethics Commission
2004233 Universidad Pontificia Bolivariana, Calle 78 B No. 72 A 109
2004238 Institutional Review Board, Ann & Robert H. Lurie Children's Hospital of Chicago, 25 East Chicago Avenue, Chicago, Illinois
2004241 Cincinnati Children's Hospital Institutional Review Board, 3333 Burnet Avenue | MLC 7040 | Cincinnati, OH 45229
2004281 MetroHealth Institutional Review board, 2500 MetroHealth Drive, Cleveland Ohio 44109
2004294 State Institution Academician O.M Lukyanova Institute of Pediatrics, obstetrics and gynecology of national academy of medical sciences of Ukraine, 8 P.Mayborody str Kyiv, 04050
2004295 CORPORACIÓN CIENTÍFICA PEDIÁTRICA, BIOMEDICAL RESEARCH ETHICS COMMITTEE, Calle 5 B5 No. 37 bis - 28
2004296 Ministerio de Salud,
Servicio de Salud Valdivia, Scientific Ethics Committee, V. Pérez Rosales 560 - Edificio Prales - Oficina 307 - Piso 3
2004300 Servicio de Salud Metropolitano Norte,
esearch Ethics Committee, 272,Calle Maruri 8380000 Independencia Metropolitana de Santiago
2004304 UNIVERSIDAD CES, Calle 10A No. 22 - 04 El Poblado
2004310 Creighton University office of the provost Research Compliance, 2500 California Plaza Omaha, NE 68178-0001
2004322 Communal Non-Commercial enterprise of Kharkiv Regional Council regional Children's clinical hospital, 5 Ozeryanska st Kharkiv, 61093
2004338 Odesa Regional State administration, department of health, communal enterprise, Odesa regional Children's clinical hospital, 3 Ac Vorobiov st, Odes-31, 65031
2004341 Medical University of South Carolina, 179 Ashley Ave, Charleston, SC 29425
2004351 MUHC Centre for Applied Ethics, 5100, boul. de Maisonneuve Ouest, 5th floor, Office 576, Montréal, Québec, H4A 3T2

2004359 Ethikkommission der Landesärztekammer Rheinland-Pfalz Deutschhausplatz 3 55116 Mainz
2004365 MUHC Centre for Applied Ethics, 5100, boul. de Maisonneuve Ouest, 5th floor, Office 576, Montréal, Québec, H4A 3T2
2004372 COMITÉ DE ÉTICA EN INVESTIGACIÓN VIT, Calle 24 N° 3-02 este
2004391 University of Nebraska Medical Center, 42nd and Emile Streets, Omaha, NE 68198, 402-559-4000
2004396 COMITATO ETICO DELLA FONDAZIONE POLICLINICO UNIVERSITARIO AGOSTINO GEMELLI IRCCS UNIVERSITÀ CATTOLICA DEL SACRO CUORE
2004400 Research Ethics Committee of the Health Sciences Department of the Universidad del Norte, Apartados Aéreos 1569 - 51820, Km. 5 vía Puerto Colombia
2004404 Federico Gomez Children's hospital of Mexico, National Institute of Health research office
2004616 Stony Brook University, Health Sciences Center Room 031, Stony Brook, NY 11794-8111
2004623 UBC C&W Research Ethics Board A2-141A, 950 West 28th Avenue Vancouver, BC V5Z 4H4
2004626 Soroka University Medical Center, Itzhak Rager Blv. Beer Sheva 8458900
2004632 Japanese Red cross Maebashi Hospital IRB 138-Asakuramachi, Maebashi-Shi Gunma
2004633 Ethics Commission at Communal Institution Dnipro City Children's Clinical Hospital No 5 of Dnipro City Council, 5 ivana Akinfiieva st, Dnipro 49027 Ukraine
2004648 Independent Ethics Committee for Clinical Pharmacology Trials, Drug and Pharmacology Studies Foundation, LA FUNDACIÓN DE ESTUDIOS FARMACOLOGICOS Y DE MEDICAMENTOS, Pte. J. E. Uriburu 774 1º Piso Ciudad Autónoma de Buenos Aires (C1027AAP)
Argentina
2004658 Nationwide Children's IRB, Nationwide Children’s Hospital, 700 Childrens Drive, Columbus, OH 43205
2004660 Yokosuka Kyosai Hospital IRB, 1-16 Yonegahamadori, Yokosuka Kanagawa
2004662 The University Of Tennessee, Health Science Centre Institutional Review Board, 910 Madison Avenue, Suite 600, Memphis, TN 38163
2004667, Conjoint Health Research Ethics Board, Research Services Office, 2500 University Drive, NW, Calgary AB T2N 1N4
2004668 Jimbo Orthopedic Surgery, Institutional Review Board, 5-38-41, Honcho Koganei-shi, Tokyo
2004669 State Social Enterprise, HOSPITAL MENTAL DE ANTIOQUIA, [Antioquia Psychiatric Hospital], Calle 38 55-310 Bello-Colombia
2004670 NHO Okayama Medical Center IRB, Kita-ku Tamasu 1711-1, Okayama-shi, Okayama-Ken, Japan
2004671 Kawasaki Municipal Hospital Institutional Review Board, 12-1, Shinkawa-dori, Kawasaki-ku, Kawasaki-shi, Kanagawa
2004672 Laniado Hospital, 16, deuteronomy haim st., kiryat sanz, netanya, 42150
2004678 Marshfield Clinic Research Institiute Institutional Review Board, 1000N, Oak Ave, Marshfield, WI 54449-5790
2004681 Human Research Ethics Committee, Fundación Hospital Infantil Universitario de San José, Carrera 52 No. 67 A-71 PBX: 4377540
2004687 Fukuyama City Hospital Institutional Review Board, 5-23-1 Zao-cho, Fukuyama-shi, Hiroshima
2004688 KKR Sapporo Medical Center IRB, 6-3-40 Hiragishi 1-jo Toyohira-ku, Sapporo-shi, Hokkaido
2004708 EMORY UNIVERSITY Institutional Review Board, 201 Dowman Dr, Atlanta, GA 30322, United States"
2004747, 2004749 Navajo Nation Human Research Review Board, Navajo Division of Health, P. O. Box 1390, Window Rock, AZ 86515
2004748 Johns Hopkins Bloomberg School Of Public Health, Institutional Review Board Office, 615 N. Wolfe Street / Room E1100 Baltimore, Maryland 21205-2179"
2004768 Samsung Medical Center Institutional Review Board, (06351) 81 Irwon-Ro Gangnam-gu. Seoul, Korea
2004769 Yonsei University Health system, Severance Hospital, Institutional review Board, Yonsei-ro 50-1, Seodaemun-gu, Seoul, 03722
2004797 human research Protection Program of Korea University medical Center 123 Jeokgeum-ro (Gojan-dong) Danwon-gu, Ansan-si, Gyeonggi-do, 15355
2004798 Inha University Hospital Institutional Review Board, 27 Inhang-ro, Jung-gu, Incheon
2004800 Yonsei University Gangnam Severance Hospital, IRB, 2nd Floor, 235 Dogok-ro, Gangnam-gu, Seoul 06230
2005029 Fukui-ken Saiseikai Hospital Institutional Review Board, 7-1 Funabashi, Wadanaka-cho, Fukui-shi, Fukui-Ken
2005030 Institutional Review Board of Okayama City General Medical Center Okayama City Hospital, 3-20-1 Kitanagaseomotemachi, Kita-ku, Okayama-shi, Okayama
2005031 Local Independent Administrative Corporation, Hiroshima City Hospital Organization, Hiroshima City Hiroshima Citizens Hospital Institutional Review Board, 7-33 Motomachi, Naka-ku, Hiroshima-shi, Hiroshima
2005032, 2005034 Review Board of Human Rights and Ethics for Clinical Studies Institutional Review Board 13-2 Ichibancho, Chiyoda-ku, Tokyo,
2005033 Aijinkai Takatsuki General Hospital IRB, 1-3-13 Kosobe-cho, Takatsuki, Osaka
2005035 Japanese Red Cross Shizuoka Hospital Institutional Review Board, 8-2 Otemachi, Aoi-ku, Shizuoka-shi, Shizuoka
2005036 JA Shizuoka Kosei Hospital Institutional Review Board, 23 Kitabancho, Aoi-ku, Shizuoka-shi, Shizuoka
2005037 Hiroshima Red Cross Hospital & Atomicbomb Survivors Hospital Institutional Review Board, 1-9-6 Sendamachi, Naka-ku, Hiroshima-shI
2005038 NHO Shikoku Medical Center for Children and Adults Institutional Review Board, 2-1-1, Senyucho, Zentsuji-shi, Kagawa, Japan
2005039 Daido Hospital Institutional Review Board, 9 Hakusuicho, Minami-ku, Nagoya, Aichi
2005049 Nagoya Ekisaikai Hospital IRB, 4-66 Shonen-Cho, Nakagawa-ku, Nagoya-Shi, Aichi
2004272, 2004044 Multicentricka eticka komise IKEM a TN,Videnska 800, Praha,140 59
2004402, 2004116 Etikprövningsmyndigheten,Box 2110,SE-750 02 Uppsala,SE-750 02
2004249, 2004298 Ethikkommission der Medizinischen Universität Graz,Auenbruggerplatz 2, Graz,8036
2004373, 2004327 Child and Adolescent Health Service (HREC), Office 5E, Perth Children's Hospital,15 Hospital Avenue Nedlands,6009
2004401, 2004399, Ethics Committee for Multicenter Trials,8 Damyan Gruev Str., Sofia,1303

2004887, 2004896 Hospital District of Southwest Finland, Joint Municipal Authority, Ethics Committee, Turku University Hospital, T-Hospital, 6th Floor, Board meeting room A 607
2004217, 2004109, 2004216 Wits Health Consortium,31 Princess of Wales Terrace,Parktown Johannesburg, 2193
2004212, 2004106, 2004214, 2004336 Ethical Council at the MoH of RF,3 Rakhmanovsky Pereulok, Moscow,127994
2004335, 2004405, 2004048, 2004105 Northern B Health and Disability Ethics Committee,20 Aitken Street, Ministry of Health, Ethics Department, Reception - Ground Floor, Thorndon, Wellington,6011
2004034, 2004108, 2004110, 2004712 Pharma Ethics Independant Research Erthics committee,123 Amcor Road,Lyttelton Manor Pretoria,0157
2004395, 2004204, 2004277, 2004710 Lithuanian Bioethics Committee,Algirdo g. 31, Vilnius,LT-03219
2004355, 2004384, 2004682, 2004689, 2004383 NRES Committee South Central - Berkshire,South West REC Centre,Level 3, Block B Bristol,BS1 2NT
2004273, 2004045, 2004046, 2004099, 2004047, 2004274 Research Ethics Committee of the National Institute for Health Development,Hiiu 42, Tallinn,11619
2004380, 2004199, 2004331, 2004202, 2004198, 2004302 Ethics Committee for Clinical Trials of Medicinal Products,Aizkraukles street 21 - 113, Riga,LV1006
2004867, 2004868, 2004869, 2004870, 2004871, 2004872 Dr Jose Renan Esquivel Children's hospital, Panama Ave, Balboa, Calle 34 Research Bioethics Committee
2004033, 2004112, 2004113, 2004114, 2004115, 2004218, 2004219, 2004311, 2004333, 2004344, 2004363, 2004369, 2004382, 2004385, 2004406, 2004675, 2004407, 2005603 Hospital Universitario Clinico San Carlos,Puerta G - Planta 4ª Norte,C/ Profesor Martin Lagos, s/n Madrid, 28040
2004674, 2004371, 2004049, 2004334, 2004206, 2004381, 2004205, 2004208, 2004350, 2004305 Komisja Bioetyczna przy Okregowej Izbie Lekarskiej w Rzeszowie,ul. Jana Dekerta 2, Rzeszów, 35-030
2004629, 2004231, 2004270, 2004299, 2004320, 2004398, 2004320 O.L.V. Ziekenhuis,Moorselbaan 164, Aalst,9300
2004303, 2004234,
2004325, 2004339,
2004343, 2004639,
2004324 Ethics Committee for Clinical Trials,8, Damyan Gruev Str.,, Sofia,1303
2004654, 2004100, 2004232, 2004374, 2004378, 2004646, 2004653, 2004676, 2005602 Comité de Protection des Personnes Ile de France VIII,Hôpital Ambroise Paré, 9 avenue Charles de Gaulle Boulogne Billancourt, 92100
2004742, 2004741, 2004313, 2004743, 2004611, 2004312, 2004644 Varsinais-Suomen sairaanhoitopiiri Eettinen toimikunta ,Kiinamyllynkatu 4-8,PL 52 Turku, 20520

Note that full information on the approval of the study protocol must also be provided in the manuscript.

Field-specific reporting

Please select the one below that is the best fit for your research. If you are not sure, read the appropriate sections before making your selection.

☒ Life sciences ☐ Behavioural & social sciences ☐ Ecological, evolutionary & environmental sciences

For a reference copy of the document with all sections, see nature.com/documents/nr-reporting-summary-flat.pdf

Life sciences study design

All studies must disclose on these points even when the disclosure is negative.

Sample size

The sample size of 1,500 is necessary based on advice from the US FDA requesting that 1,000 preterm infants be exposed to nirsevimab in this Phase 2b study. This sample size has approximately > 99% power to detect 70% relative risk reduction, assuming a placebo group medically attended RSV LRTI incidence of 8%. Power calculations are based on Poisson regression model with robust variance (Zou Am J Epidemiol 2004;159:702-706) comparing nirsevimab 50 mg versus placebo, with 2-sided, $\alpha = 0.049$ (due to 0.001 alpha spend at the interim analysis; refer to Interim Analysis Section 4). The 70% relative risk reduction assumption is based on a placebo-controlled study in Native American infants in which there was 87% relative reduction in the incidence of RSV hospitalization (11.3% placebo; 1.5% motavizumab; $p < 0.001$) and 71% relative reduction in the incidence of outpatient RSV LRTI (10.0% placebo; 2.9% motavizumab; $p < 0.001$) in infants who received motavizumab prophylaxis (O'Brien et al. Lancet Infect Dis 2015;15:1398-1408) In order to evaluate risk, a sample size of 1,000 subjects exposed to nirsevimab will provide a 90% probability of observing at least one AE if the true event rate is 0.2%; if no AEs are observed, this study provides 95% confidence that the true event rate is < 0.3%.

With 3000 subjects, MELODY had at least 99% power for the primary efficacy endpoint. Analysis of the primary efficacy endpoint based on the 1490 subjects randomised prior to the pause of the enrolment due to the COVID-19 pandemic, still allowed the study to be sufficiently powered. More specifically, the sample size of approximately 1500 subjects in the Primary Cohort has at least 99% power to detect a 70% RRR, assuming an 8% incidence of MA RSV LRTI in the placebo group. Power calculations were based on a Poisson regression model with robust variance (Zou Am J Epidemiol 2004;159:702-706) comparing nirsevimab versus placebo, with 2-sided, $\alpha = 0.05$. The assumption of 8% incidence is supported both by literature (Paramore et al. Pediatr Pulmonol 2010;45:578-584) and the observed placebo incidence rate (9.6%) in Study 3. The 70% RRR assumption is based on Study 3 in which there was a 70% RRR in the incidence of MA RSV LRTI (9.5% placebo, 2.6% nirsevimab; $p < 0.001$) and 79% RRR in the incidence of MA RSV LRTI with hospitalisation (4.1% placebo, 0.8% nirsevimab; $p < 0.001$) in subjects who received nirsevimab prophylaxis. In addition, the assumption is supported by a placebo-controlled study in Native American term infants in which there was a 71% relative reduction in the incidence of outpatient RSV LRTI (10.0% placebo, 2.9% motavizumab; $p < 0.001$) and 87% relative reduction in the incidence of RSV hospitalisation (11.3% placebo, 1.5% motavizumab; $p < 0.001$) in infants who received motavizumab prophylaxis (O'Brien et al. Lancet Infect Dis 2015;15:1398-1408). In the event that the incidence rate in the placebo group decreased due to the impact of the COVID-19 pandemic (eg, social distancing), the sample size of 1500 provided at least 90% power to detect a 70% RRR if the placebo incidence rate is 4% or higher.

Data exclusions	No data were excluded from the analysis
Replication	Results obtained from validated assays were not replicated. Serum neutralization results (non-validated assay) was performed in duplicate.
Randomization	An interactive web response system was used for randomization to a treatment group and assignment of blinded investigational product kit numbers in both studies. Pooling was based on the treatment groups generated at randomization as no further treatment was administered.
Blinding	The subject/legal representative, investigators and site staff were blinded with regard to the treatment received.

Reporting for specific materials, systems and methods

We require information from authors about some types of materials, experimental systems and methods used in many studies. Here, indicate whether each material, system or method listed is relevant to your study. If you are not sure if a list item applies to your research, read the appropriate section before selecting a response.

Materials & experimental systems

n/a	Involved in the study
☐	☒ Antibodies
☐	☒ Eukaryotic cell lines
☒	☐ Palaeontology and archaeology
☒	☐ Animals and other organisms
☐	☒ Clinical data
☒	☐ Dual use research of concern

Methods

n/a	Involved in the study
☒	☐ ChIP-seq
☒	☐ Flow cytometry
☒	☐ MRI-based neuroimaging

Antibodies

Antibodies used	For the validated assays:<br>Mouse anti-RSV F primary (Millipore, Cat. No. MAB858-1); Clone 133-1H, used at 1:30,000 dilution.<br>Peroxidase conjugated goat anti-mouse secondary antibody (Life Technologies, Cat. No. A16072) used at 1:30,000 dilution.<br><br>In non-validated assays:<br>Mouse anti-RSV F primary (Millipore, Cat. No. MAB8262); Clone 133-1H, used at 1:5000 dilution.<br>Dako polyclonal goat anti mouse IgG-HRP Cat. No. P0447 used at 1:4000 dilution.
Validation	Further details of the antibodies can be found on the manufacturers website:<br>https://www.merckmillipore.com/GB/en/product/Anti-RSV-Antibody-fusion-protein-all-type-A-B-strains-clone-133-1H,MM_NF-MAB858-1#anchor_COA<br>https://www.thermofisher.com/antibody/product/Goat-anti-Mouse-IgG-H-L-Cross-Adsorbed-Secondary-Antibody-Polyclonal/A16072<br><br>https://www.merckmillipore.com/GB/en/product/Anti-RSV-Antibody-fusion-protein-all-type-A-B-strains-clone-133-1H,MM_NF-MAB8262<br>https://www.agilent.com/en/product/specific-proteins/elisa-kits-accessories/goat-anti-mouse-immunoglobulins-hrp-affinity-isolated-2717109

Eukaryotic cell lines

Policy information about cell lines and Sex and Gender in Research

Cell line source(s)	Hep-2 cells (ATCC, Cat. No Hep-2 CCL-23)
Authentication	A cryovial of the Hep-2 cells was sent to a 3rd party vendor (IDEXX) for PCR analysis. The samples were confirmed to be of human origin and no mammalian inter-species contamination was detected. Cells were authenticated using PCR to identify the presence of the following markers: amelogenin, CSF1PO, D13S317, D16S539, D5S818, TH01, TPOX and vWA. The genetic profiles for the samples were identical to the genetic profiles reported for each cell line.
Mycoplasma contamination	A cryovial of the Hep-2 cells was sent to a 3rd party vendor (IDEXX) to be tested for mycoplasma and other contaminates. Report showed that cell line was negative for Mycoplasma sp., bacterial growth, and fungal growth.
Commonly misidentified lines<br>(See ICLAC register)	Hep-2 cells. The cells were fully authenticated prior to use.

Clinical data

Policy information about clinical studies

All manuscripts should comply with the ICMJEguidelines for publication of clinical research and a completedCONSORT checklist must be included with all submissions.

Clinical trial registration	Phase 2b: NCT02878330; MELODY: NCT03979313
Study protocol	Phase 2b: https://clinicaltrials.gov/ct2/show/NCT02878330. MELODY: the protocol is not yet publicly available as the study is ongoing
Data collection	Phase 2b was conducted at 164 sites in 23 countries across the globe between November 3, 2016 (study start date) and December 6, 2018 (actual study completion date).<br>The MELODY primary cohort was conducted at 160 sites in 21 countries across the globe between July 23, 2019 (study start date) and March 11, 2020 (enrolment pause due to COVID-19 pandemic) with a final estimated completion date of March 21, 2023.

Outcomes

Phase 2b:
Primary outcome measures:
Number of participants with medically attended respiratory syncytial virus (RSV) confirmed lower respiratory tract infection (LRTI) [Time frame: from Day 1 through Day 151]
The determination of medically attended RSV LRTI is based on objective clinical LRTI criteria and RSV test results obtained from analysing the respiratory secretions using a validated RSV real time reverse transcriptase-polymerase chain reaction (RT-PCR) assay for the detection of RSV A or RSV B subtypes. Criteria for LRTI included documented physical exam findings of rhonchi, rales, crackles, or wheeze and any of the following: increased respiratory rate at rest (for age 2 months: ≥60 breaths/min; 2-6 months: ≥50 breaths/min; and for >6 months - 2 years, ≥40 breaths/min), or hypoxemia (in room air - oxygen saturation <95% at altitudes ≤1800 meters or <92% at altitudes >1800 meters), or clinical signs of severe respiratory disease or dehydration secondary to inadequate oral intake due to respiratory distress (need for intravenous fluid).
Secondary outcome measures:
1. Number of participants hospitalized due to RSV confirmed LRTI [Time frame: from Day 1 through Day 151]
An RSV hospitalization is defined as either 1) a respiratory hospitalization with a positive RSV test within 2 days of hospitalization (primary) or 2) new onset of respiratory symptoms in an already hospitalized child, with an objective measure of worsening respiratory status and positive RSV test (nosocomial).
2. Number of participants with treatment emergent adverse events (TEAEs) and treatment emergent serious adverse events (TESAEs) [Time frame: from Day 1 through Day 361]
3. Number of participants with adverse events of special interest (AESIs) and new onset chronic diseases (NOCDs) [Time Frame: From Day 1 through Day 361]
4. Serum concentration of nirsevimab [time frame: Days 91, 151, and 361]
5. Elimination half-life (t1/2) of nirsevimab [Time frame: Day 91 through Day 361]
6. Number of participants with positive anti-drug antibodies (ADA) to nirsevimab [Time frame: Days 91, 151, and 361]

MELODY:
Primary outcome measures:
1. Incidence of medically attended LRTI due to RT-PCR confirmed RSV [Time frame: 150 days post-dose]
The incidence of RSV LRTI (inpatient and outpatient) 150 days post dose will be based on RSV test results (performed centrally via RT-PCR) and objective clinical LRTI criteria and will be presented by treatment group. The relative risk reduction of nirsevimab over placebo in preventing RSV LRTI will be estimated from model.
Secondary outcome measures:
1. Incidence of hospitalization due to RT-PCR confirmed RSV [Time frame: 150 days post-dose]
The incidence of RSV hospitalization 150 days post dose will be presented by treatment group. The relative risk reduction of nirsevimab over placebo in preventing RSV hospitalization will be estimated from model.
2. Safety and tolerability of nirsevimab as assessed by the occurrence of all TEAEs and TESAE [Time frame: 360 days post-dose]
Other safety assessments will include the occurrence of AESIs and NOCDs.
3. Single-dose serum concentrations of nirsevimab [Time frame: 360 days post-dose]
Nirsevimab serum concentration levels will be assessed by mean serum concentration of nirsevimab at pre-specified timepoints and tabulated by treatment group.
4. Incidence of ADA to nirsevimab in serum [Time frame: 360 days post-dose]
The incidence of ADA to nirsevimab will be assessed and summarized by percentage of subjects that are ADA positive by treatment group.
